# Host Transcriptional Meta-signatures Reveal Diagnostic Biomarkers for *Plasmodium falciparum* Malaria

**DOI:** 10.1093/infdis/jiae041

**Published:** 2024-01-25

**Authors:** Nágila Isleide Silva, Pedro Felipe Loyola Souza, Bárbara Fernandes Silva, Simone Gonçalves Fonseca, Luiz Gustavo Gardinassi

**Affiliations:** Departamento de Biociências e Tecnologia, Instituto de Patologia Tropical e Saúde Pública, Universidade Federal de Goiás, Goiânia, Brazil; Departamento de Biociências e Tecnologia, Instituto de Patologia Tropical e Saúde Pública, Universidade Federal de Goiás, Goiânia, Brazil; Departamento de Biociências e Tecnologia, Instituto de Patologia Tropical e Saúde Pública, Universidade Federal de Goiás, Goiânia, Brazil; Departamento de Biociências e Tecnologia, Instituto de Patologia Tropical e Saúde Pública, Universidade Federal de Goiás, Goiânia, Brazil; Departamento de Biociências e Tecnologia, Instituto de Patologia Tropical e Saúde Pública, Universidade Federal de Goiás, Goiânia, Brazil; Departamento de Enfermagem Materno-Infantil e Saúde Pública, Escola de Enfermagem de Ribeirão Preto, Universidade de São Paulo, Ribeirão Preto, Brazil

**Keywords:** immune response, gene expression, biomarker, bacterial infection, cerebral malaria

## Abstract

**Background:**

Transcriptomics has been used to evaluate immune responses during malaria in diverse cohorts worldwide. However, the high heterogeneity of cohorts and poor generalization of transcriptional signatures reported in each study limit their potential clinical applications.

**Methods:**

We compiled 28 public data sets containing 1556 whole-blood or peripheral blood mononuclear cell transcriptome samples. We estimated effect sizes with Hedge's *g* value and the DerSimonian-Laird random-effects model for meta-analyses of uncomplicated malaria. Random forest models identified gene signatures that discriminate malaria from bacterial infections or malaria severity. Parasitological, hematological, immunological, and metabolomics data were used for validation.

**Results:**

We identified 3 gene signatures: the uncomplicated Malaria Meta-Signature, which discriminates *Plasmodium falciparum* malaria from uninfected controls; the Malaria or Bacteria Signature, which distinguishes malaria from sepsis and enteric fever; and the cerebral Malaria Meta-Signature, which characterizes individuals with cerebral malaria. These signatures correlate with clinical hallmark features of malaria. Blood transcription modules indicate immune regulation by glucocorticoids, whereas cell development and adhesion are associated with cerebral malaria.

**Conclusions:**

Transcriptional meta-signatures reflecting immune cell responses provide potential biomarkers for translational innovation and suggest critical roles for metabolic regulators of inflammation during malaria.

## Teaser

We performed integrative analyses of multiple cohorts spanning >1500 transcriptome samples to uncover gene signatures that discriminate malaria from healthy controls, enteric fever, and sepsis or that distinguish cerebral malaria from other clinical phenotypes.

Malaria causes thousands of deaths worldwide and accurate diagnosis combined to effective treatment are critical to control *Plasmodium* transmission. Malaria diagnosis is a straightforward process when trained microscopists and clinicians are available. Nevertheless, biomarkers are desired to predict outcomes of infection, severity, and therapeutical success, and to discriminate malaria from other febrile diseases. Because inflammation causes clinical manifestations of malaria, biomarkers underlying the immune response can provide indicators of disease, point to molecular mechanisms of pathogenesis, and reveal novel pharmacological targets [1].

Diverse transcriptomic-based studies reported biological processes, pathways, and genes associated with the human immune response during malaria [2–14]. However, age, sex, geographic location, parasite species, sample type, profiling technology, and several other confounding factors account for the high variability of gene expression in response to *Plasmodium* infection [15]. The poor generalizability of the findings from individual studies limits their clinical translation.

Integration of transcriptomics data obtained from multiple independent cohorts is a powerful approach to identify biomarkers with diagnostic and prognostic applications [16, 17]. To overcome the limitations of analyses using single cohorts, we compiled 28 public transcriptomic data sets that span >1500 samples to perform multicohort analyses. We explored this compendium with meta-analyses and random forest models, which revealed gene signatures that differentiate uncomplicated *P. falciparum* malaria from controls or culture-confirmed bacterial infections; or characterize patients with cerebral malaria (CM). Importantly, the results suggest significant activity of immunometabolic regulators during malaria.

## METHODS

### Data Compilation and Processing

Public microarray and RNA sequencing data from humans infected with *Plasmodium* were downloaded from the Gene Expression Omnibus (GEO), ArrayExpress and European Nucleotide Archive repositories. Each gene expression data set contained ≥5 whole-blood or peripheral blood mononuclear cell (PBMC) samples for case patients and controls. The cohorts included patients with uncomplicated malaria (UM) and participants from controlled human malaria infections (CHMIs). We also downloaded data sets including samples from patients with severe malarial anemia (SMA) or CM. Some data sets included samples from individuals with asymptomatic infection, presymptomatic infection, enteric fever (EF), community-acquired pneumonia (CAP), and chronic obstructive pulmonary disease (COPD). Additional data sets included samples from individuals with EF or sepsis. Uninfected controls included samples obtained from healthy individuals, obtained before CHMI (baseline), or obtained after treatment for malaria (Tables 1 and 2).

**Table 1.** Data Sets Used for Meta-analyses and Extended Validations

Accession ID^a^	Technology	Platform^b^	Sample Type	Infection Type	*Plasmodium* Species	Samples, No.									Reference
						UM	Healthy Controls	Baseline	Treated	Extended Validation					
										Mixed^c^	Asymp	Presymp	EF/CAP	COPD	
Discovery data sets uMMS															
GSE34404	Array	GPL10558	WB	Natural	*falciparum*	94	61	…	…	…	…	…	…	…	[4]
GSE5418	Array	GPL96	PBMC	Natural/CHMI	*falciparum*	15	…	22	…	…	…	22	…	…	[2]
GSE7000	Array	GPL4857	WB	Natural	*falciparum*	11	19	…	…	…	…	…	21	…	[18]
GSE119150	Array	GPL15207	WB	Natural	*falciparum*	6	6	…	…	…	…	…	…	…	[9]
GSE52166	RNA-seq	GPL11154	WB	Natural	*falciparum*	25	26	…	…	…	17	29	…	…	[11]
GSE156791	RNA-seq	GPL16791	WB	Natural	*falciparum*	36	36	…	…	…	…	…	…	…	[19]
GSE181179	RNA-seq	GPL11154	PBMC	Natural	*falciparum*	6	6	…	…	…	5	…	…	…	[14]
Validation data sets uMMS															
GSE1124	Array	GPL96	WB	Natural	*falciparum*	5	5	…	…	…	5	…	…	…	[10]
GSE64338	Array	GPL6244	WB	Natural	*falciparum*	17	…	…	19	…	…	…	…	…	[5]
GSE15221	Array	GPL6102	PBMC	Natural	*falciparum*	14	…	…	14	…	…	…	…	…	[3]
GSE94916	Array	GPL20844	PBMC	Natural	*falciparum*	6	5	…	…	…	…	…	5	5	[12]
GSE50957	RNA-seq	GPL11154	WB	CHMI	*falciparum*	5	2	5	…	…	…	…	…	…	[7]
PRJEB45911	RNA-seq	GPL20301	WB	Natural	*falciparum*	12	7	…	…	…	25	…	…	…	[20]
Data sets used only in extended validation															
GSE132050	Array	GPL17586	WB	CHMI	*falciparum*	…	…	8	…	8	…	32	…	…	[13]
GSE97158	RNA-seq	GPL11154	WB	CHMI	*falciparum*	…	…	10	…	…	…	20	…	…	[21]
GSE172481	RNA-seq	GPL24676	WB	CHMI	*falciparum*	…	…	28	…	29	…	117	…	…	[22]
GSE67184	RNA-seq	GPL16791	PBMC	CHMI	*vivax*	6	…	6	…	…	…	…	…	…	[6]
GSE144792	RNA-seq	GPL24676	WB	Natural	*vivax*	33	31	…	…	…	…	…	…	…	[23]

Abbreviations: Asymp, asymptomatic infection; CAP, community-acquired pneumonia; CHMI, controlled human malaria infection; COPD, chronic obstructive pulmonary disease; EF, enteric fever; ID, identification number; PBMCs, peripheral blood mononuclear cells; Presymp, presymptomatic infection; RNA-seq, RNA sequencing; UM, uncomplicated malaria; uMMS, uncomplicated Malaria Meta-Signature; WB, whole blood.

^a^Accession IDs are from Gene Expression Omnibus (GEO) database, except for PRJEB45911, which is deposited at the European Nucleotide Archive.

^b^Platforms according to GEO database.

^c^Mixed samples, including both asymptomatic infection and symptomatic malaria.

Robust multi-array average–normalized data were downloaded for Affymetrix arrays, and quantile normalization was used for other array platforms. Probes were mapped to genes using biomaRt R package v. 2.56.1 [30]. We used the “MaxMean” method of the collapseRows function from Weighted Correlation Network Analysis R package v. 1.72.1 to summarize probes. Data were log_2_ transformed. Normalized RNA sequencing data were downloaded from GEO. For some data sets, raw data were downloaded from the NCBI Sequence Read Archive using the SRA toolkit v. 3.0.1. Quality control was performed with FastQC v. 0.11.9, and adapter sequence and trimming were performed with fastp v. 0.23.0. Trimmed reads were mapped to the human genome (GRCh38.p13) and quantified using the Rsubread package v. 2.16.0 [31]. Data were normalized to counts per million reads mapped and log_2_-transformed using edgeR v. 3.42.4 [32], and gene annotations were performed with biomaRt v. 2.56.1 [30].

### Transcriptional Meta-analysis of Uncomplicated *P. falciparum* Malaria

We split the data sets into 7 discovery and 6 validation cohorts. Discovery cohorts included 193 individuals with UM and 176 healthy controls. Validation cohorts included 59 individuals with UM and 57 controls (Table 1). Extended validations included samples from individuals with asymptomatic infection, presymptomatic infection, mixed asymptomatic and symptomatic infection, *Plasmodium vivax* malaria, EF, CAP, and COPD (Table 1). We used the MetaIntegrator R package v. 2.1.3 [33] to analyze discovery cohorts by calculating the Hedge’s adjusted *g* value as the effect size for each gene in each data set, which were latter combined with DerSimonian-Laird random-effects model. The Benjamini-Hochberg false discovery rate (FDR) was used to correct for multiple hypothesis testing. To account for unbalanced sample sizes and different sample types (PBMCs or whole blood), we used a leave-one-out approach that removed one data set at a time from the meta-analysis. An FDR-adjusted *P* value threshold of <.001, an effect size of 1.6-fold, and presence in ≥6 data sets identified 16 genes that were significant at all iterations.

### Identification of Diagnostic Signatures With Random Forest models

To identify a signature that discriminates malaria from bacterial infections, we used the COmbat CO-Normalization Using conTrols (COCONUT) method from the metaIntegrator R package to combine microarray data sets acquired from whole-blood samples from patients with *P. falciparum* UM, SMA, CM, sepsis, or EF and respective uninfected controls (Table 2). This resulted in a “superset” of 714 samples and 6209 genes. Discovery cohorts included arrays from Illumina platforms, while validation cohorts included other platforms. To identify a signature that discriminates CM from other clinical phenotypes, we also combined arrays from patients with UM, SMA, or CM and uninfected controls, using the Combat method (Table 2). This resulted in a “superset” of 190 samples and 11 070 genes. Discovery cohorts included arrays from diverse platforms and the one independent validation cohort (E-MTAB-6413) was acquired with RNA sequencing and normalized separately. Discovery cohorts were used for feature selection using random forest analysis and built-in feature selection according to implementations in MetaboAnalyst 5.0 [34]. Selected models were refined by forward search method in the MetaIntegrator R package.

**Table 2.** Data Sets Used for Random Forest Analyses

Accession ID^a^	Technology	Platform^b^	Sample Type	Infection Type	Disease	Samples, No.								Reference
						UM	SMA	CM	Healthy Controls	Baseline	Treated	Sepsis	EF	
Discovery data sets MoBS														
GSE34404	Array	GPL10558	WB	Natural	Malaria	94	…	…	61	…	…	…	…	[4]
GSE117613	Array	GPL10558	WB	Natural	Malaria	…	17	17	12	…	…	…	…	[8]
GSE25504	Array	GPL6947	WB	Natural	Sepsis	…	…	…	35	…	…	24	…	[24]
GSE137340	Array	GPL10558	WB	Natural	Sepsis	…	…	…	12	…	…	23	…	…
GSE113866	Array	GPL10558	WB	Natural	EF	…	…	…	47	…	…	…	31	[16]
GSE112958	Array	GPL10558	WB	CHI	EF	…	…	…	78	…	…	…	50	[16]
Validation data sets MoBS														
GSE7000	Array	GPL4857	WB	Natural	Malaria/EF	11	…	…	19	…	…	…	21	[18]
GSE119150	Array	GPL15207	WB	Natural	Malaria	6	…	…	6	…	…	…	…	[9]
GSE1124	Array	GPL96	WB	Natural	Malaria	5	5	5	5	…	…	…	…	[10]
GSE64338	Array	GPL6244	WB	Natural	Malaria	17	…	…	…	…	19	…	…	[5]
GSE33341	Array	GPL571	WB	Natural	Sepsis	…	…	…	43	…	…	51	…	[25]
Discovery data sets cMMS														
GSE117613	Array	GPL10558	WB	Natural	Malaria	…	17	17	12	…	…	…	…	[8]
GSE1124	Array	GPL96	WB	Natural	Malaria	5	5	5	5	…	…	…	…	[10]
GSE116306	Array	GPL16699	PBMCs	Natural	Malaria	6	4	6	…	…	…	…	…	[26]
GSE72058	Array	GPL6244	WB	Natural	Malaria	…	…	98	…	…	…	…	…	[27]
GSE33811	Array	GPL6244	WB	Natural	Malaria	5	…	5	…	…	…	…	…	[28]
Validation data set cMMS														
E-MTAB-6413	RNA-seq	GPL16791	WB	Natural	Malaria	25	…	20	…	…	…	…	…	[29]

Abbreviations: CHI, controlled human infection; CM, cerebral malaria; cMMS, cerebral Malaria Meta-Signature; EF, enteric fever; ID, identification number; MoBS, Malaria or Bacteria Signature; PBMCs, peripheral blood mononuclear cells; RNA-seq, RNA sequencing; SMA, severe malarial anemia; UM, uncomplicated malaria; WB, whole blood.

^a^Accession IDs are from the Gene Expression Omnibus (GEO) database, except for E-MTAB-6413, which is deposited at the ArrayExpress database.

^b^Platforms according to the GEO database.

### Blood Transcription Module Meta-analyses

Gene expression in each data set was reduced to the activity of 346 blood transcription modules (BTMs) [35] using the tmod R package [36], which performs principal component analysis (PCA) for each BTM in each data set, retaining the PC1 score as the module activity. We used the MetaIntegrator package to perform BTM-driven meta-analyses or random forest analyses ,as described in the previous sections. For meta-analysis, the significance threshold was set at *P* < .05 (FDR adjusted). Random forest analyses were also applied to BTMs to identify differences between malaria and bacterial infections or to determine malaria severity. The forward search method in the MetaIntegrator R package was used to refine selected signatures.

### Meta-signature scores

The uncomplicated Malaria Meta-Signature (uMMS), Malaria or Bacteria Signature (MoBS), cerebral Malaria Meta-Signature (cMMS), and CM BTM scores were calculated as the *z* score of the difference between the geometric mean of up-regulated genes/BTMs and the geometric mean of down-regulated genes/BTMs.

### Additional Validation Data

We obtained parasitemia levels for the data sets GSE117613, GSE34404, GSE119150, GSE52166, and PRJEB45911 at the GEO repository. In addition the following were available for the data set GSE117613 at GEO: levels of *P. falciparum* histidine-rich protein 2 (PfHRP2); hematological data, including whole-blood cell, neutrophil, lymphocyte, monocyte, platelet, and red blood cell (RBC) counts; quantification of mean corpuscular volume (MCV), hemoglobin, erythropoietin (EPO), heme oxygenase, and bilirubin; and cytokine and chemokine data, including quantification of interleukin 1β, 1RA, 6, 8, 10, and 12, interferon γ, CXCL10, CCL3, CLL4, and tumor necrosis factor. These data were used in correlation analyses with identified transcriptional signatures. We also downloaded metabolomics data available for the data set GSE156791, which are deposited at the Metabolomics Workbench repository (under accession no. ST001400). Metabolomics data were normalized by the mean, and all data were log_2_ transformed. These data were used in correlation analyses to obtain functional insights.

### Statistical Analyses and Visualization

We used *t* tests, paired *t* tests, analysis of variance with the Bonferroni multicomparison test, and Spearman correlations to evaluate statistical significance where applicable. Meta-analyses of correlations between gene signatures and parasitemia were performed using Fisher’s method. Receiver operating characteristic (ROC) curve analysis was used to test gene signatures, and the area under the curve (AUC) was used as a performance metric. Brier scores were used to evaluate accuracy of probabilistic predictions (Supplementary Table 1). Heat maps, forest plots, and ROC analysis were performed with the MetaIntegrator R package v. 2.1.3. Bubble plots were generated with the ggplot2 R package v. 3.4.4.

## RESULTS

### Identifying a Conserved Gene Signature for Uncomplicated *P. falciparum* Malaria With Meta-analysis

**Figure 1.**
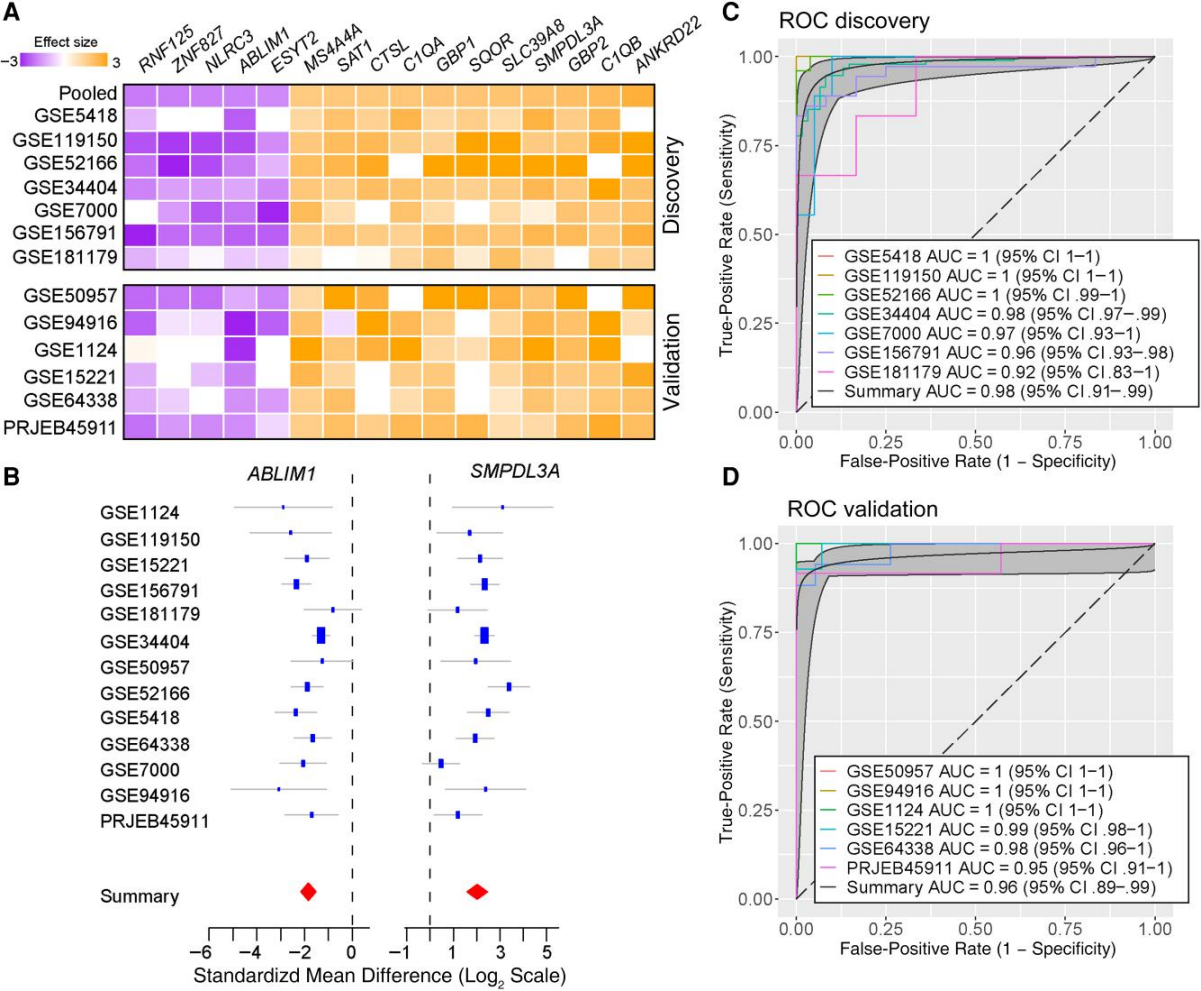
Uncomplicated malaria induces a conserved transcriptional signature in blood leukocytes. *A*, Heat map of the effect sizes of genes composing the uncomplicated Malaria Meta-Signature in discovery (n = 369) and validation (n = 116) cohorts. *B*, Representative forest plots of one down-regulated gene, *ABLIM1,* and one up-regulated gene, *SMPDL3A*. The standardized mean difference in the x-axis is computed as the log_2_-transformed Hedge’s adjusted *g* value. The standard error of the mean for each study is inversely proportional to the size of blue rectangles, and 95% confidence intervals (CIs) are represented by whiskers. Red diamonds reflect the combined mean difference for each gene, and their width represents the 95% CI. *C, D,* Receiver operating characteristic (ROC) curves comparing individuals with uncomplicated *Plasmodium falciparum* malaria and controls in discovery (*D*) and validation (*E*) cohorts. Abbreviation: AUC, area under the curve.

To identify a unified transcriptional signature characterizing the human immune response during *P. falciparum* UM, we downloaded 13 gene expression data sets in public repositories (Table 1). The cohorts include individuals with *P. falciparum* UM due to natural infection or CHMI. We used 7 discovery cohorts in a meta-analysis that resulted in 11 up-regulated and 5 down-regulated genes (*P* < 1.5 × 10^−8^; FDR < 6.6 × 10^−7^; effect size, 1.6-fold), whose differential expression was highly reproducible in the validation cohorts (*P* < .004; FDR < 0.05) (Figure 1*A*). This gene set was termed the uMMS. Effect sizes of *ABLIM1* and *SMPDL3A* are shown for all UM cohorts in Figure 1*B*; see Supplementary Figure 1 for the remaining genes. ROC analysis based on the uMMS resulted in a summary AUC of 0.98 (95% confidence interval [CI], .91–.99) for discovery (Figure 1*C*) and 0.96 (.89–.99) for validation cohorts (Figure 1*D*).

Infection with *Plasmodium* can be asymptomatic or cause severe complications and death. We tested whether the uMMS could distinguish between different clinical phenotypes. A score based on the uMMS distinguished UM from asymptomatic infection (Supplementary Figure 2), but it was not associated with severity (Supplementary Figure 3). The uMMS score increased in samples from individuals with presymptomatic malaria in only 2 of 5 cohorts (Supplementary Figure 4) but clearly distinguished mixed asymptomatic/symptomatic malaria from control samples (Supplementary Figure 4*B* and 4*C*). The uMMS displayed lower performance for discriminating *P. vivax* malaria from control samples (Supplementary Figure 5).

Malaria can be confounded with other inflammatory diseases, especially febrile infections [16, 18]. The uMMS showed significant ability to discriminate between UM and EF, CAP, and COPD (Supplementary Figure 6).

### Defining a Diagnostic Signature That Differs Between Malaria and Bacterial Infections

**Figure 2.**
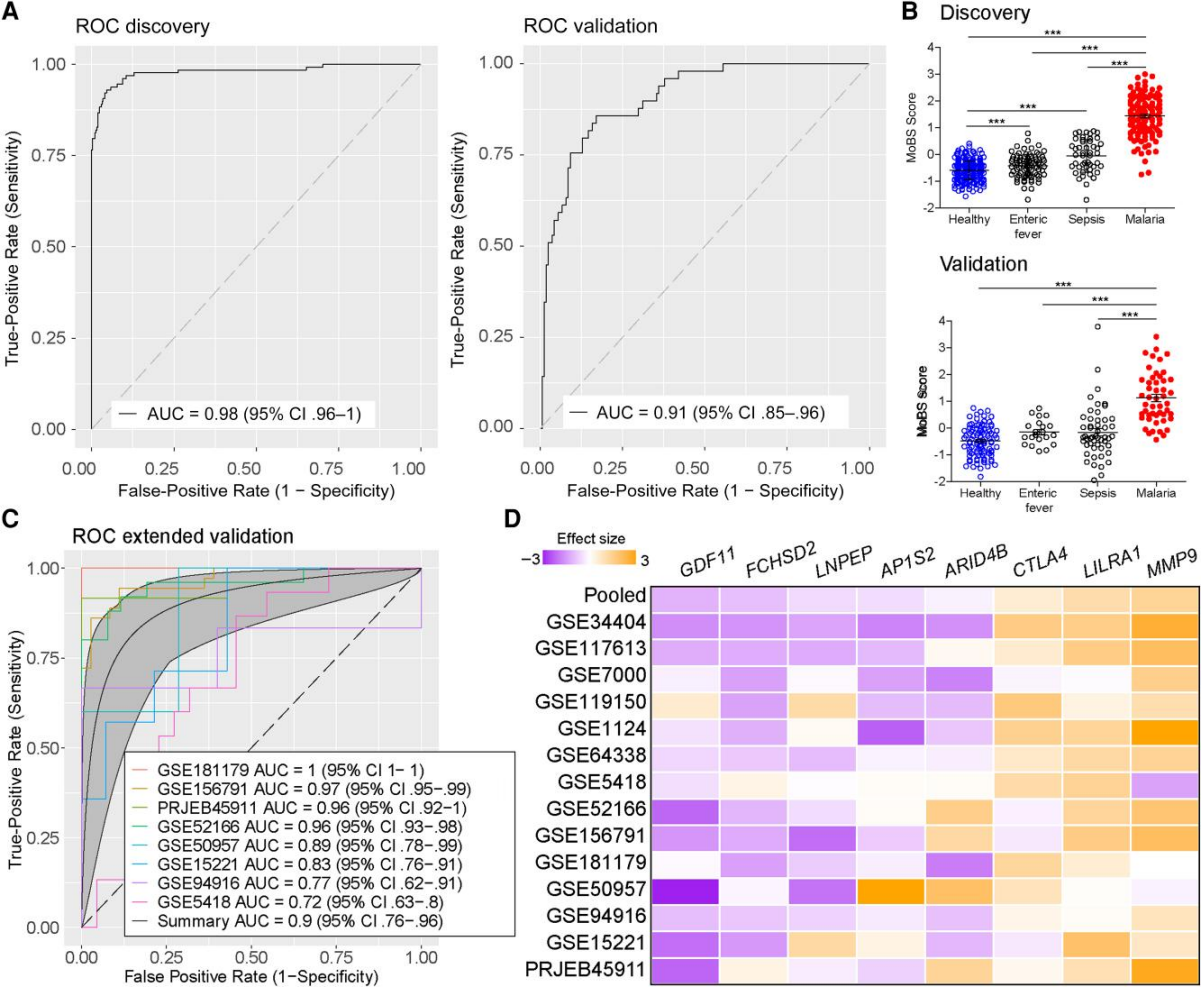
Random forest analysis identifies a signature discriminating malaria or bacterial infections. *A*, Receiver operating characteristic (ROC) curves comparing individuals with *Plasmodium falciparum* malaria and individuals with enteric fever, sepsis, and controls in discovery and validation cohorts using the Malaria or Bacteria Signature (MoBS). Abbreviations: AUC, area under the curve; CI, confidence interval. *B*, MoBS score comparing individuals with *P. falciparum* malaria and individuals with enteric fever or sepsis and controls in discovery and validation cohorts. ***P < .001. *C*, ROC curves comparing individuals with uncomplicated malaria and controls using MoBS. *D*, Heat map of the effect sizes of genes composing MoBS in individuals with malaria compared with controls (n = 531). MoBS scores were compared using analysis of variance followed by the Bonferroni multicomparison test. Error bars represent means with standard errors of the mean.

Meta-analysis relies on the heterogeneity of diverse cohorts containing samples from relevant phenotypes (eg, malaria and controls; malaria and EF). Therefore, we were unable to use this strategy to find a signature discriminating malaria from other bacterial diseases such as sepsis. To overcome this issue, we merged multiple cohorts from patients with malaria, EF, sepsis, and controls into a “superset” and split them into discovery and validation cohorts (Table 2). We applied random forest analysis in a 2-class approach, using malaria as cases and all the other conditions as controls, as described elsewhere [16]. The analysis resulted in diverse models containing different numbers of classifier genes. We chose a signature containing 25 genes, which was submitted to a forward search approach within the MetaIntegrator package. We identified an 8-gene signature that discriminates malaria from EF, sepsis, and controls with high-performance in discovery and validation cohorts (Figure 2*A*).

The MoBS score showed significant differences between malaria and the other groups in discovery and validation cohorts (Figure 2*B*). It also distinguished UM from controls in 8 independent data sets, with a summary AUC of 0.9 (95% CI, .76–.96) (Figure 2*C*). Effect sizes for genes composing MoBS revealed higher variability compared with uMMS, but pooled effect sizes are in accordance with random forest results (Figure 2*D*). The MoBS score differed in 1 of 5 cohorts of presymptomatic malaria (Supplementary Figure 7), but it did not differ between controls and mixed asymptomatic/symptomatic samples (Supplementary Figure 7*B* and 7*C*) or *P. vivax* UM (Supplementary Figure 8). MoBS score differed between SMA and CM in one data set included in the discovery cohort but not in an independent data set (Supplementary Figure 9).

### Transcriptional Meta-signature Characterizing Cerebral Malaria

**Figure 3.**
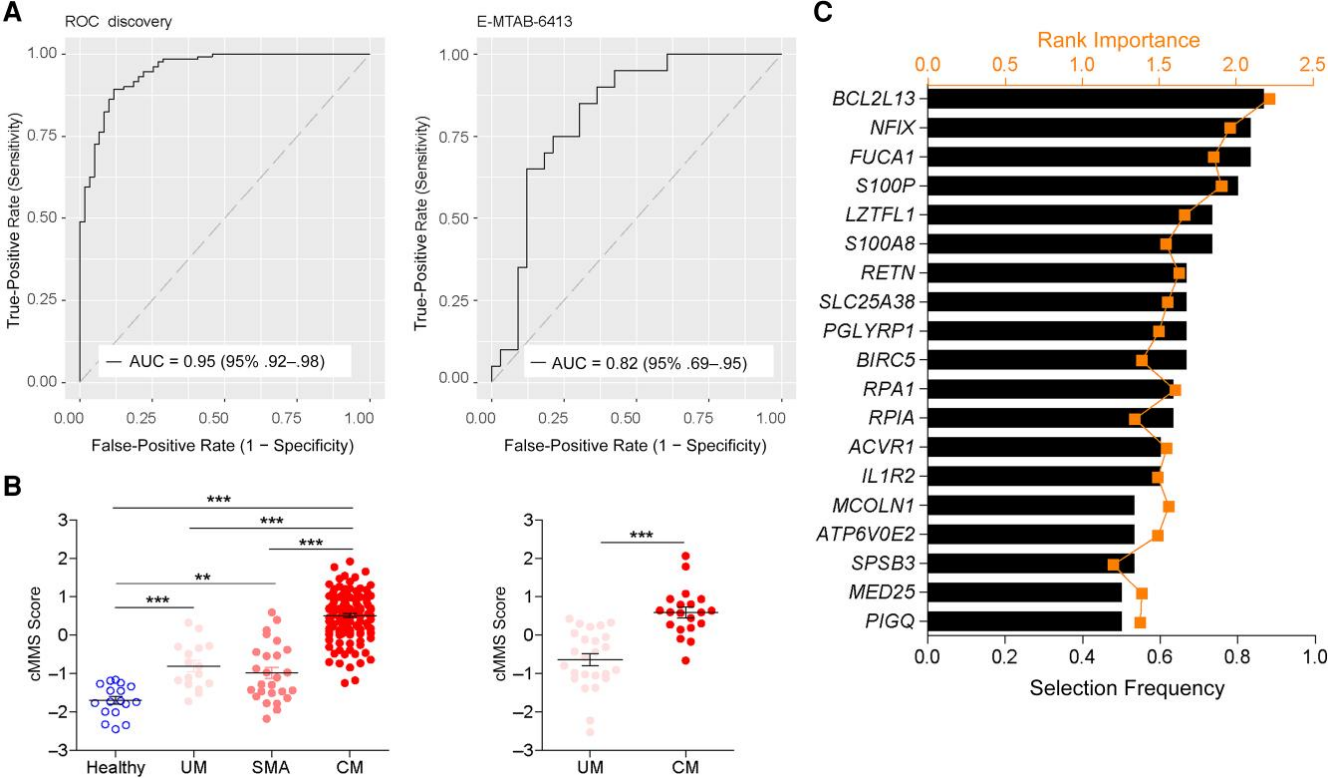
Cerebral malaria (CM) is characterized by a systemic transcriptional signature. *A*, Receiver operating characteristic (ROC) curves comparing individuals with CM and individuals with uncomplicated malaria (UM) or severe malarial anemia (SMA) and controls in discovery and validation cohorts using the cerebral Malaria Meta-Signature (cMMS). Abbreviations: AUC, area under the curve; CI, confidence interval. *B*, cMMS score comparing individuals with CM and individuals with UM or SMA and controls in discovery and validation cohorts. *C*, Genes composing the cMMS ordered by selection frequency and rank importance in the random forest model. cMMS scores were compared using analysis of variance followed by the Bonferroni multicomparison test. Error bars represent means with standard errors of the mean. **P < .01; ***P < .001.

Because neither uMMS nor MoBS could reliably distinguish malaria severity, we also used random forest analysis to derive a cMMS. For that, we merged microarray data sets containing samples from individuals with UM, SMA, and CM and from controls into a “superset” of 190 samples that was used as discovery cohort (Table 2). Random forest analysis using a 2-class classifier differentiating CM from the rest resulted in a model with 25 genes that was manually curated by removing genes whose contribution was not relevant for final AUC. We identified a 19-gene signature that distinguished CM from other phenotypes, with AUCs of 0.95 in the discovery cohort and 0.82 in an independent validation data set (E-MTAB-6413) (Figure 3*A*). The cMMS score differed between CM and all other groups, but it also differed between controls and UM or SMA (Figure 3*B*). Selection frequency and rank importance of genes included in the cMMS are shown in Figure 3*C*. cMMS distinguished UM from controls for most cohorts but displayed reduced discriminatory power for 3 of them (Supplementary Figure 10*A*). It also demonstrated reduced ability to simultaneously discriminate between malaria and EF, sepsis, and controls (Supplementary Figure 10*B*).

### Correlation Between Meta-signatures and Hallmarks of Clinical Malaria

*Plasmodium* infection causes RBC disruption as a direct function of parasite replication, which triggers the inflammatory response. Meta-analyses of Spearman rank correlation between the signature scores and parasitemia revealed significant positive correlations for all of them, with uMMS displaying the highest meta-coefficient (Supplementary Figure 1^1^*A*). We also evaluated the associations between the identified signature scores and other parasitological, hematological, and cytokine and chemokine data available for the data set GSE117613 [8]. There were positive correlations with PfHRP2 and hemoglobin levels (Supplementary Figure 11*A* and 11*B*) and negative associations with RBC, MCV, and platelet counts for all of them, but only the cMMS score was significantly correlated with EPO levels (Supplementary Figure 1^1^*B*). The 3 signature scores were correlated positively with plasmas abundance of interleukin 1RA, 6, 8, and 10, tumor necrosis factor, CCL2, CCL4, and CXCL10 (Supplementary Figure 11*C* and 11*D*).

### BTM Activity Reflecting Conserved Human Immune Responses to Malaria

**Figure 4.**
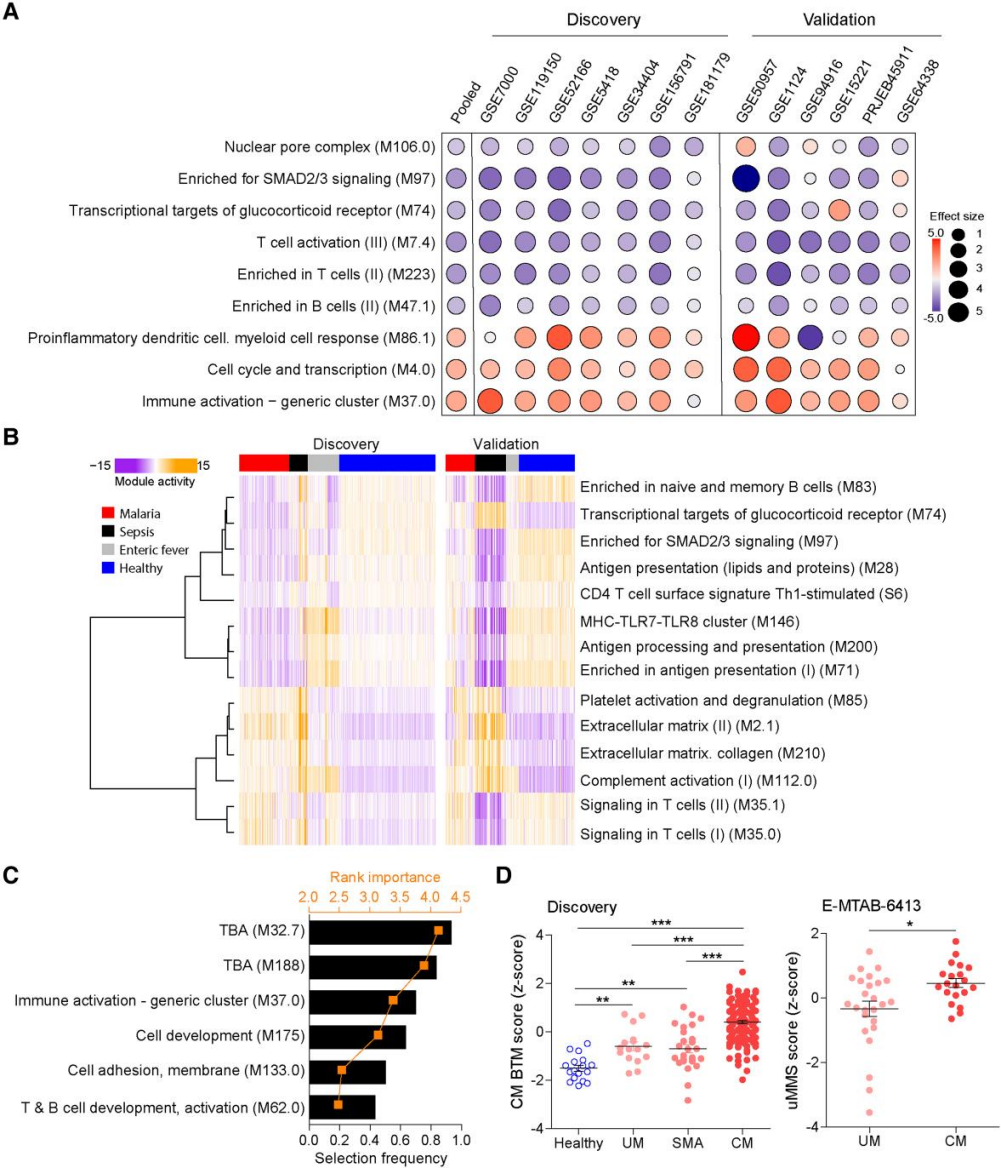
Blood transcription module (BTM) underlying host responses to *Plasmodium falciparum* malaria. *A*, Bubble plots of the effect sizes of significant BTMs in discovery and validation cohorts, using meta-analysis of uncomplicated *P. falciparum* malaria and controls shown in Table 1. The bubble size and color scale are proportional to the standardized mean difference (effect size) between individuals with malaria and controls. *B*, Heat map of BTM activity in individuals with *P. falciparum* malaria and individuals with enteric fever or sepsis and controls in discovery and validation cohorts shown in Table 2. *C*, BTMs differing between individuals with cerebral malaria (CM) and individuals with uncomplicated malaria (UM) or severe malarial anemia (SMA) and controls, ordered by selection frequency and rank importance in the random forest model. *D*, CM BTM signature score differentiating CM from other clinical phenotypes in discovery and validation cohorts. *P < .05; **P < .01; ***P < .001. Abbreviations: MHC, major histocompatibility complex; TBA, to be assigned; Th1, T-helper 1; SMAD, Mother against Dpp (MAD) homology (MH) domain transcription factor; TLR, Toll-like receptor; uMMS, uncomplicated Malaria Meta-Signature.

The unbiased BTM framework captures gene expression activity that translates into immunological and metabolic processes in the human blood [35]. We performed a BTM-driven meta-analysis to identify conserved activity of gene modules during UM. Meta-analysis with 7 discovery cohorts (Table 1), using a leave-one-out and forward search approach, resulted in a set of 6 down-regulated and 3 up-regulated BTMs related to nuclear pore complex, SMAD2/3 signaling, transcriptional targets of glucocorticoid receptor (GR), T-cell activation, B cells, proinflammatory myeloid cells, cell cycles, and immune activation (Figure 4*A*). ROC analysis using this BTM set revealed summary AUCs of 0.97 (95% CI, .85–1) for discovery cohorts and 0.95 (.8–.99) for validation cohorts (Supplementary Figure 1^2^*A*). This BTM set displayed poor discriminatory performance in both natural infection and CHMI in *P. vivax* malaria cohorts (Supplementary Figure 1^2^*B*).

To gather further insights into the transcriptional profiles that underlie the immune responses to infection, we also used BTM-driven random forest models to identify conserved modules that differ between malaria and other conditions, such as EF, sepsis, and healthy controls. We used the MoBS discovery superset (Table 2) in random forest analysis to select 14 BTMs that discriminate malaria from other groups with relevant performance in discovery and validation cohorts (Supplementary Figure 1^2^*C*). These BTMs are related to T and B lymphocytes, transcriptional targets of GR, SMAD2/3 signaling, antigen presentation, platelets, extracellular matrix, and complement (Figure 4*B*). We also used the cMMS discovery “superset” in BTM-driven random forest analysis and a forward search method to unveil 6 BTMs, mostly related to immune activation, cell development, and adhesion (Figure 4*C*). This BTM set discriminates CM from other groups in the discovery cohort and validation data set (Supplementary Figure 1^2^*D*). Calculation of a CM BTM score revealed significant differences between CM and UM or SMA in the discovery cohort and validation data set (Figure 4*D*).

### Enzymatic Activity and Metabolite Signaling During Malaria Suggested by Conserved Host Gene Expression

**Figure 5.**
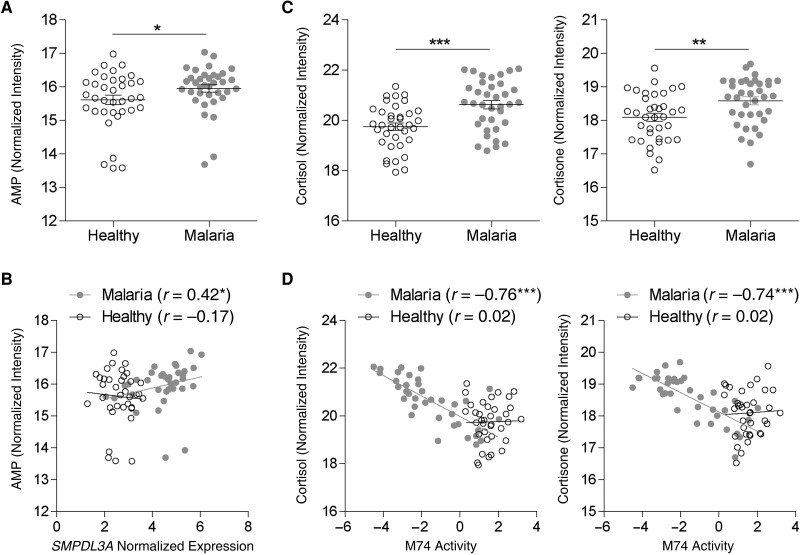
Functional validation via integration with metabolomics data. *A*, Comparison of the relative abundance of 5´-adenosine monophosphate (AMP) in the plasma before infection (healthy individuals) and during *Plasmodium falciparum* malaria. *B*, Associations between *SMPDL3A* expression and AMP before (healthy) and during *P. falciparum* malaria. *C*, Comparison of the relative abundance of cortisol and cortisone in the plasma before (healthy) and during *P. falciparum* malaria. *D*, Associations between M74 activity and cortisol, and cortisone before (healthy) and during *P. falciparum* malaria. Data were analyzed using paired *t* tests and Spearman rank correlation. *P < .05; **P < .01; ***P < .001.

The genes composing the signatures and the BTMs associated with malaria are presumably translated into proteins and enzymes involved in metabolism and cell signaling in blood leukocytes. To gain insights into the functional activity of genes encoding enzymes and BTMs associated with metabolism, we integrated pairwise transcriptomics and metabolomics data available for the data set GSE156791. *SMPDL3A* encodes sphingomyelin phosphodiesterase acidlike 3A, an enzyme that hydrolyses nucleotide diphosphate and triphosphate (eg, adenosine diphosphate, adenosine triphosphate [ATP]) into nucleotide monophosphate (eg, adenosine monophosphate [AMP]) (Supplementary Figure 13*A*) [37]. *SMPDL3A* expression was consistently up-regulated in the blood of individuals with malaria when compared with uninfected controls in diverse cohorts (Figure 1*B*). Of note, the relative levels of AMP were increased in the plasma (Figure 5*A*) and were correlated positively with *SMPDL3A* expression during malaria but not before infection (Figure 5*B*).

The BTM transcriptional targets of GR (M74) showed reduced activity during UM (Figure 4*A* and Supplementary Figure 14*A*). At the same time, the relative abundance of glucocorticoids, such as cortisol and cortisone, increased in plasma with malaria (Figure 5*C*). Glucocorticoids diffuse through cell membranes and interact with GR in the cytoplasm, which is transported to the nucleus and regulates the expression of transcriptional targets of GR (Supplementary Figure 13*B*) [38]. Strikingly, M74 activity exhibited strong negative correlations with the abundance of cortisol and cortisone only after clinical malaria (Figure 5*D*). Those associations are driven by genes such as *CD3E*, *ZC3H12D*, *HLA-DOA*, and *MEF2C* (Supplementary Figure 14*B*).

## DISCUSSION

Malaria diagnosis can be accomplished without difficulty by trained microscopists and clinicians in structured settings. However, there have been emerging concerns about resistance to rapid diagnostic tests in highly endemic areas, which motivates searches for alternatives [39]. Differentiating symptomatic malaria from febrile illness with coincidental parasitemia is also critical, as severe bacterial infection of patients asymptomatically infected with *P. falciparum* can confound diagnosis and misguide appropriate treatment. Here, we identified transcriptional biomarkers of disease caused by *P. falciparum* that differentiate UM or CM or that discriminate malaria from bacterial infection with significant performance. Importantly, we identified associations between different data types that suggest relevant immunometabolic activity during malaria.

We used both meta-analyses and data set merging followed by random forest analysis to identify gene meta-signatures, all of which present advantages and limitations. The uMMS displays the best performance for distinguishing *Plasmodium* infection from uninfected controls, including *P. vivax* malaria. However, it had a moderate performance for distinguishing malaria from bacterial infection, and it could not discriminate malaria severity. Therefore, the uMMS represents a broad transcriptional signature that has significant overlap with gene expression induced by other inflammatory conditions. The MoBS scores exhibits good performance for discriminating malaria from EF, sepsis, and controls simultaneously, but the magnitude of scores and not whether the gene is expressed in a determined condition is what drives those differences. MoBS also seems to be specific for clinical *P. falciparum* malaria, shown by reduced performance for differentiating presymptomatic and mixed samples from controls. However, as it was generated with whole-blood samples, it displayed lower generalizability for data acquired from PBMCs. The cMMS identified samples from CM with good performance both in discovery and validation cohorts, but it also showed lower generalizability for discriminating malaria from other conditions.

Anemia is a typical clinical manifestation of malaria that is caused in part by parasite replication. Positive associations with parasitemia and levels of PfHRP2 concomitant with negative associations with RBC counts and MCV support the significant role of genes composing the different signatures. Furthermore, cMMS had the strongest association with MCV and was significantly associated with EPO, which promotes RBC production and function and plays an important role in the pathogenesis of CM [40]. The data suggest that the expression of selected genes fluctuates according to the interactions between host and *Plasmodium*, whereby parasite growth causes RBC destruction, which, in turn, activates the inflammatory response. Associations with cytokines and chemokines in the plasma corroborate this hypothesis.

We identified positive association between *SMPDL3A* expression in leukocytes and relative levels of AMP in the plasma during malaria. Sphingomyelin phosphodiesterase acidlike 3A (encoded by *SMPDL3A*) is an enzyme secreted by macrophages, which displays nucleotide phosphodiesterase activity against nucleotide triphosphates, including ATP [37]. These data suggest that in response to *Plasmodium* infection, leukocytes secrete SMPDL3A to convert ATP into AMP to regulate the inflammatory signaling of purinergic receptors [41]. Moreover, SMPDL3A is a cyclic guanosine monophosphate–AMP degrading enzyme that restricts cGAS-STING signaling [42] and may contribute to regulate type I interferon responses during malaria [43]. Single-cell transcriptomics of PBMCs from *P. falciparum* UM demonstrate that type I interferon responses are activated in all cell types, suggesting it is linked to induction of immunoregulatory networks during malaria [44].

We found significant modulation and associations between transcriptional targets of GR (M74) and the abundance of cortisol and cortisone, suggesting that, in addition to pregnenolone [19], glucocorticoids also regulate immune cell responses during malaria, but this hypothesis requires further validation using other methods. BTM analyses also revealed significant associations of modules underlying immune activation, cell development, and adhesion during CM. Mouse models suggest that the dynamics of leukocyte development, activation, and adhesion to brain vasculature can be linked to the severity of CM [45]. However, adherent properties of leukocytes during CM may also affect other organs to promote pathology [46].

Both gene-level and BTM-level analyses suggest that the transcriptional signatures identified in this study are mostly associated with *P. falciparum* malaria, because they fail to characterize individuals infected with *P. vivax* with the same performance. Those differences might be driven by higher parasite densities during *P. falciparum* malaria, which is supported by significant associations between gene signatures and parasitemia. However, specific technical limitations may also influence the results, including sequencing depth and coverage for both data sets with *P. vivax* malaria.

In conclusion, we identified host transcriptional responses with potential for translational innovation that also contribute to a better understanding of the human immune response to *P. falciparum* malaria.

## Supplementary Data

Supplementary materials are available at *The Journal of Infectious Diseases* online (http://jid.oxfordjournals.org/). Supplementary materials consist of data provided by the author that are published to benefit the reader. The posted materials are not copyedited. The contents of all supplementary data are the sole responsibility of the authors. Questions or messages regarding errors should be addressed to the author.

## Supplementary Material

jiae041_Supplementary_Data

## Notes

***Acknowledgments***. The authors thank all participants and researchers involved in previous studies that generated and shared the data used in the current study.

***Author contributions***. L. G. G. acquired funding, designed, and supervised the study. N. I. S., P. F. L. S, B. F. S., and L. G. G. performed data collection, processing, analyses, and visualization. S. G. F. contributed critical resources and discussion. L. G. G. wrote the initial draft, and all authors revised and agreed with the final version of the manuscript.

***Financial support*.** This work was supported by the Serrapilheira Institute (grant Serra—R-2011–37433), Fundação de Apoio à Pesquisa (FUNAPE), and Conselho Nacional de Desenvolvimento Científico e Tecnológico (CNPq; research fellowship to S. G. F. and L G. G.).
