# Supplementary material for: Host Transcriptional Meta-signatures Reveal Diagnostic Biomarkers for Plasmodium falciparum Malaria
Source: J Infect Dis. 2024 Jan 25;230(2):e474–85. doi: 10.1093/infdis/jiae041 (PMC11326815; doi:10.1093/infdis/jiae041)
Supplement: jiae041_Supplementary_Data [file jiae041_supplementary_data.pdf]

**Supplementary information**

Description of datasets used in the study. Information is reported according to previous descriptions of datasets in each independent study and other details such as number of samples, profiling platform and other are described in Tables 1 and 2.

**GSE34404:** Idaghdour et al. 2012 [1] used microarray technology to evaluate gene expression in whole blood from children with uncomplicated malaria and age-matched uninfected healthy controls residing in the Republic of Benin. *P. falciparum* infection was confirmed using rapid diagnostic test and by standard blood smear.

**GSE5418:** Ockenhouse et al. 2006 [2] used microarray technology to profile gene expression from peripheral blood mononuclear cells (PBMCs) from different groups of individuals. The first group comprised adult, male and female, malaria-naïve volunteers aged 20 to 45 years residing in the United States of America that were subjected to controlled human malaria infection (CHMI) with *P. falciparum* (strain 3D7) via bites of laboratory-reared *Anopheles stephensi*. Samples were collected immediately before the mosquito challenge and at the time of diagnosis, defined by identification of a single asexual blood-stage parasite by microscopy, when those individuals were pre-symptomatic. The second group included PBMC samples from adults aged 19 to 49 years residing in Camerron, West Africa, which presented fever and symptoms of uncomplicated malaria (UM), and which had confirmed *P. falciparum* infection by standard blood smear. Uninfected, healthy malaria-naïve subjects residing in the United States of America served as controls.

**GSE7000:** Thompson et al. 2009 [3] used microarray technology to evaluate transcriptional profiles from whole blood of patients with UM, enteric fever (EF) and healthy controls residing in the Vietnam. Infection was determined by standard blood smear for malaria and blood culture for enteric fever. Authors enrolled patients with uncomplicated malaria caused mostly by *P. falciparum*. The one sample of malaria caused by *P. vivax* was excluded from the analysis. Uninfected individuals served as healthy controls.

**GSE119150:** Li et al. 2018 [4] used microarray technology to evaluate transcriptional profiles in whole blood samples from adult male Chinese patients with uncomplicated malaria, infected with *P. falciparum* in Angola. Age-matched healthy subjects were used as uninfected controls.

**GSE52166:** Tran et al. 2019 [5] used RNA sequencing (RNA-seq) technology to profile gene expression in whole blood samples from children residing in Mali, which were infected with *P. falciparum*. The samples originated from a longitudinal follow-up of over 600 adults and children, which were classified into immune subjects (asymptomatic), subjects with delayed fever (pre-

symptomatic), and with febrile disease, which was considered UM. Samples from the same subjects before infection were used as uninfected controls.

**GSE156791:** Abdrabou et al. 2021 [6] used RNA-seq technology to investigate the gene expression profiles in whole blood samples from children with UM residing in Burkina Faso. Samples were collected before and after infection with *P. falciparum*, which was detected with rapid test and confirmed with standard blood smear and PCR. Samples collected before infection were used as uninfected controls.

**GSE181179:** Studniberg et al 2022 [7] used RNA-seq technology to evaluate the transcriptional activity in PBMC samples from children and adults with uncomplicated malaria residing in Indonesia. *P. falciparum* malaria was confirmed with standard blood smear and PCR. The study also included asymptomatic *P. falciparum* infection and healthy individuals were used as uninfected controls.

**GSE1124:** Boldt et al 2019 [8] used microarray technology to profile gene expression in whole blood samples from children residing in Gabon, infected with *P. falciparum*, and classified into differing groups of severity, including asymptomatic infection, UM, severe malarial anaemia (SMA) and cerebral malaria (CM). *Plasmodium* infection was confirmed by standard blood smear. Community children were used as uninfected controls.

**GSE64338:** Subramaniam et al. 2015 [9] microarray technology to investigate the transcriptional profile in whole blood from male and female adults with UM. *P. falciparum* infection was confirmed by standard blood smear and rapid diagnostic test. Blood samples from the same subjects were collected 30 days after treatment with artemether-lumefantrine and were used as uninfected controls.

**GSE15221:** Franklin et al. 2009 [10] used microarray technology to profile transcriptional patterns in PBMCs from patients with UM residing in Brazil. *P. falciparum* infection was confirmed by standard blood smear and PCR at the time of diagnosis and after 3 – 4 weeks of treatment with mefloquine. Samples collected after treatment of each patient served as uninfected controls.

**GSE94916:** Bertrams et al. 2021 [11] used microarray technology to evaluate the transcriptional profiles in PBMC samples from adults with UM, infected with *P. falciparum*. The dataset also included samples from subjects with bacterial community acquired pneumonia (CAP) and chronic obstructive pulmonary disease (COPD). Healthy subjects were used as uninfected controls.

**GSE50957:** Tran et al. 2016 [12] used RNA-seq technology to determine the transcriptional responses in whole blood samples from female adult volunteers residing in the Netherlands. The volunteers were subjected to CHMI with NF54 *P. falciparum* via bites of laboratory-reared mosquitos. *Plasmodium*

infection was confirmed by standard blood smear. Samples collected before the challenge plus two healthy volunteers were used as uninfected controls.

**PRJEB45911:** Prah et al. 2023 [13] used RNA-seq technology to profile the gene expression in whole blood samples from children with UM residing in Ghana. *Plasmodium* infection was confirmed by standard blood smear. The dataset also included asymptomatically infected children and healthy children were used as uninfected controls.

**GSE132050:** Milne et al. 2021 [14] used microarray technology to determine the transcription patterns in whole blood samples from male and female adult volunteers residing in the United Kingdom that were subjected to CHMI with *P. falciparum* (strain 3D7) via intravenous injection of 690 infected erythrocytes. Diagnosis was defined by positive blood smear and/or parasitaemia > 500 parasites/mL quantified by qPCR and/or symptoms of malaria. This group of samples was considered mixed asymptomatic/symptomatic UM. Samples collected before infection were used as uninfected controls and samples collected along the course of the infection were considered as pre-symptomatic.

**GSE97158:** Rothen et al. 2018 [15] used RNA-seq technology to evaluate the transcriptional signatures in while blood samples from male volunteers residing in Tanzania that were submitted to CHMI with *P. falciparum* via intradermal infection of aseptic, purified, cryopreserved *P. falciparum* sporozoites. Samples collected at five and nine days after infection were considered pre-symptomatic and samples collected before infection were used as uninfected controls.

**GSE172481:** Sandoval et al. 2021 [16] used RNAseq technology to profile the transcription patterns in whole blood samples from male and female adult volunteers residing in the United Kingdom that were subjected to CHMI with *P. falciparum* (strain 3D7) via intravenous injection of approximately 1000 infected erythrocytes. Volunteers were re-challenged for two to three times and samples were collected longitudinally. Diagnosis was defined by presence of symptoms with parasitaemia > 5000 parasites/mL or parasitaemia > 10000 parasites/mL at any time, quantified by qPCR. Therefore, this group of samples was considered mixed with asymptomatic/symptomatic UM. Samples collected before infection were used as controls and samples collected along the course of the three challenges were considered pre-symptomatic.

**GSE67184:** Rojas-Peña et al. 2015 [17] used RNA-seq technology to profile the transcriptome of whole blood samples from naïve and malaria-exposed male and female adult volunteers residing in Colombia. The volunteers were subjected to CHMI with a clinical isolate of *P. vivax* via bites of laboratory-reared *Anopheles albimanus*. Samples for RNA-seq analysis were collected at the day of first detection of *P. vivax* by standard blood smear test and only samples from naïve volunteers were

used, the other group of samples containing 2 asymptomatic and 4 pre-symptomatic phenotypes. Samples collected before infection were used as uninfected controls.

**GSE144792:** Easton et al. 2020 [18] used RNA-seq technology to study the transcriptome of children infected with *P. vivax* or co-infection with *P.* vivax and soi-transmitted helminths residing in Colombia. Diagnosis was performed with thick blood smear. Only samples from children uniquely infected with *P. vivax* were analysed in our study and healthy children were used as uninfected controls.

**GSE117613:** Nallandhighal et al. 2019 [19] used microarray technology to investigate the gene expression patterns in whole blood samples from children residing in Uganda, infected with *P. falciparum*, and classified into differing groups of severity including SMA and CM. *Plasmodium* infection was confirmed by standard blood smear. Healthy community children were used as uninfected controls.

**GSE25504:** Smith et al. 2015 [20] used microarray technology to profile the transcriptional signatures in whole blood samples from neonates with sepsis residing in the United Kingdom, infected with multiple bacterial species. Infection was confirmed by blood-culture. Blood collected from healthy infants due other clinical reasons were used as controls.

**GSE137340:** Mukhopadhay et al. 2019 used microarray technology to evaluate the gene expression profiles in whole blood samples from adults with sepsis residing in India. Blood collected from healthy individuals were used as controls.

**GSE113866:** Blohmke et al. 2019 [21] used microarray technology to investigate the transcription profiles in whole blood samples from individuals with EF caused by *Salmonella enterica* serovars Typhi or Paratyphi residing in Nepal. Infection was confirmed by blood-culture. Healthy community individuals were used as controls.

**GSE112958:** Blohmke et al. 2019 [21] used microarray technology to profile the gene expression in whole blood samples from adult volunteers residing in the United Kingdom. They were subjected to controlled human infection (CHI) with S. Typhi Quailes strain and diagnosis was based on fever > 38°C sustained for 12h and/or blood culture. Healthy individuals were used as controls.

**GSE33341:** Ahn et al. 2013 [22] used microarray technology to analyse the transcriptional patters in whole blood samples from adults with sepsis with confirmed infection by blood-culture residing in the United States of America. Healthy individuals were used as controls.

**GSE116306:** Thiam et al. 2019 [23] used microarray technology to carry out transcriptome studies in PBMC samples from children and adult patients with UM, SMA and CM residing in Senegal. *P. falciparum* infection was confirmed by standard blood smear and an immunoassay detecting PfHRP2.

**GSE72058:** Feintuch et al. 2016 [24] used microarray technology to characterize the gene expression profiles in whole blood samples from 98 children with cerebral malaria with or without retinopathy residing in Malawi.

**GSE33811:** Krupka et al. 2012 [25] used microarray technology to identify transcriptional patterns in whole blood samples from children with UM and CM residing in Malawi. *P. falciparum* infection was confirmed by standard blood smear.

**E-MTAB-6413:** Lee et al. 2018 [26] used RNA-seq technology to investigate the gene expression profiles in whole blood samples from children with UM and CM residing in Gambia. *P. falciparum* infection was confirmed by standard blood smear.

**Supplementary Table 1. Brier scores for main models**

| Model | Brier score | Refer to figure |
|---|---|---|
| uMMS – Discovery | 0.05 | Figure 1c |
| uMMS – Validation | 0.07 | Figure 1d |
| MoBS – Discovery | 0.03 | Figure 2a |
| MoBS – Validation | 0.12 | Figure 2a |
| MoBS – Extended Validation | 0.10 | Figure 2c |
| cMMS – Discovery | 0.07 | Figure 3a |
| cMMS – E-MTAB-6413 | 0.21 | Figure 3a |

uMMS – uncomplicated Malaria Meta-Signature; MoBS – Malaria or Bacteria Signature; cMMS – cerebral Malaria Meta-Signature.

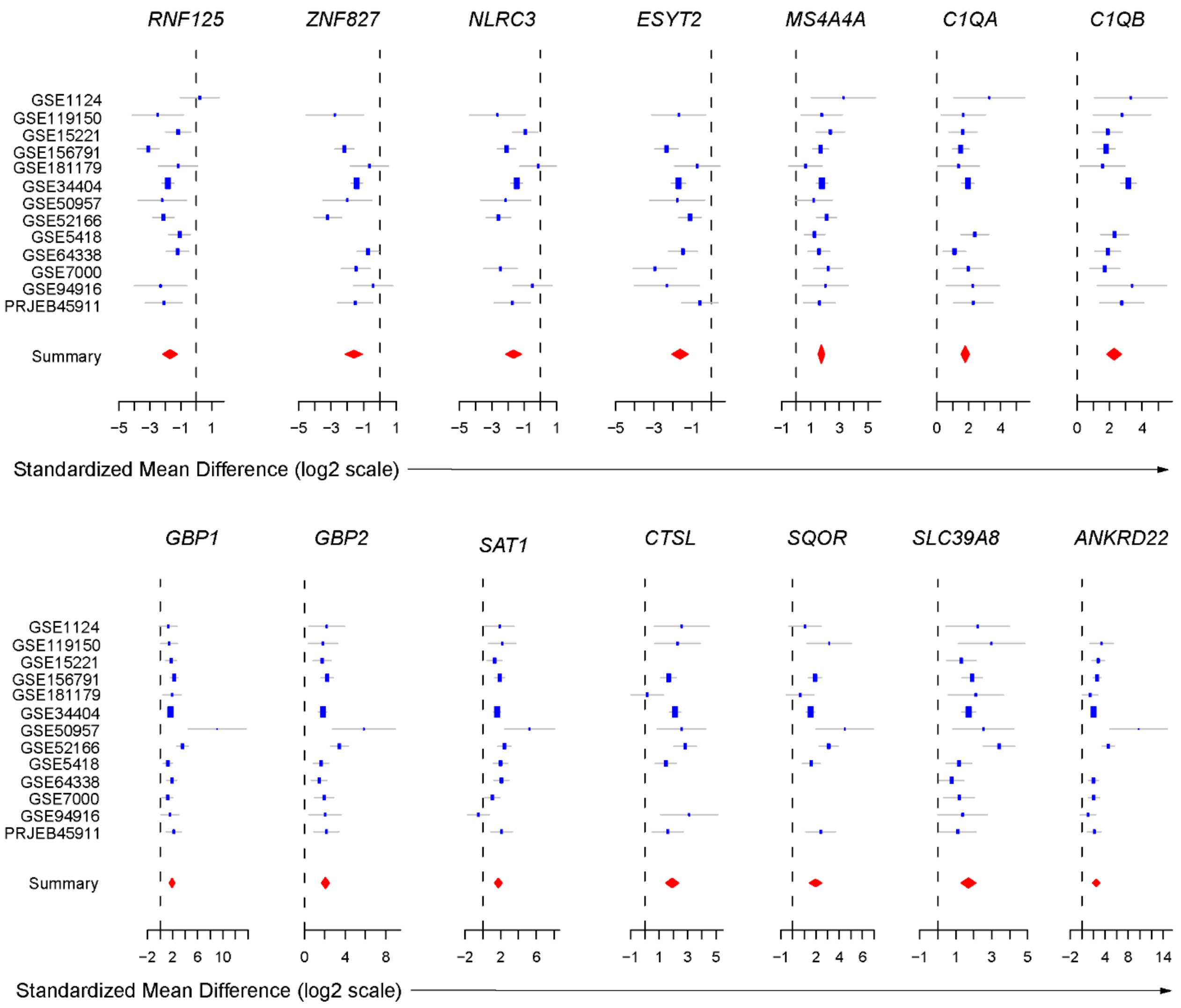


**Supplementary Figure 1 – Forest plots of genes composing the uncomplicated Malaria Meta-Signature (uMMS).** The standardized mean difference in the x axes is computed as the log2-transformed Hedges´ adjusted g. SEM for each study is inversely proportional to the size of blue rectangles and 95% confidence intervals are represented by whiskers. The red diamonds reflect the combined mean difference for each gene and their width represent the 95% confidence interval.

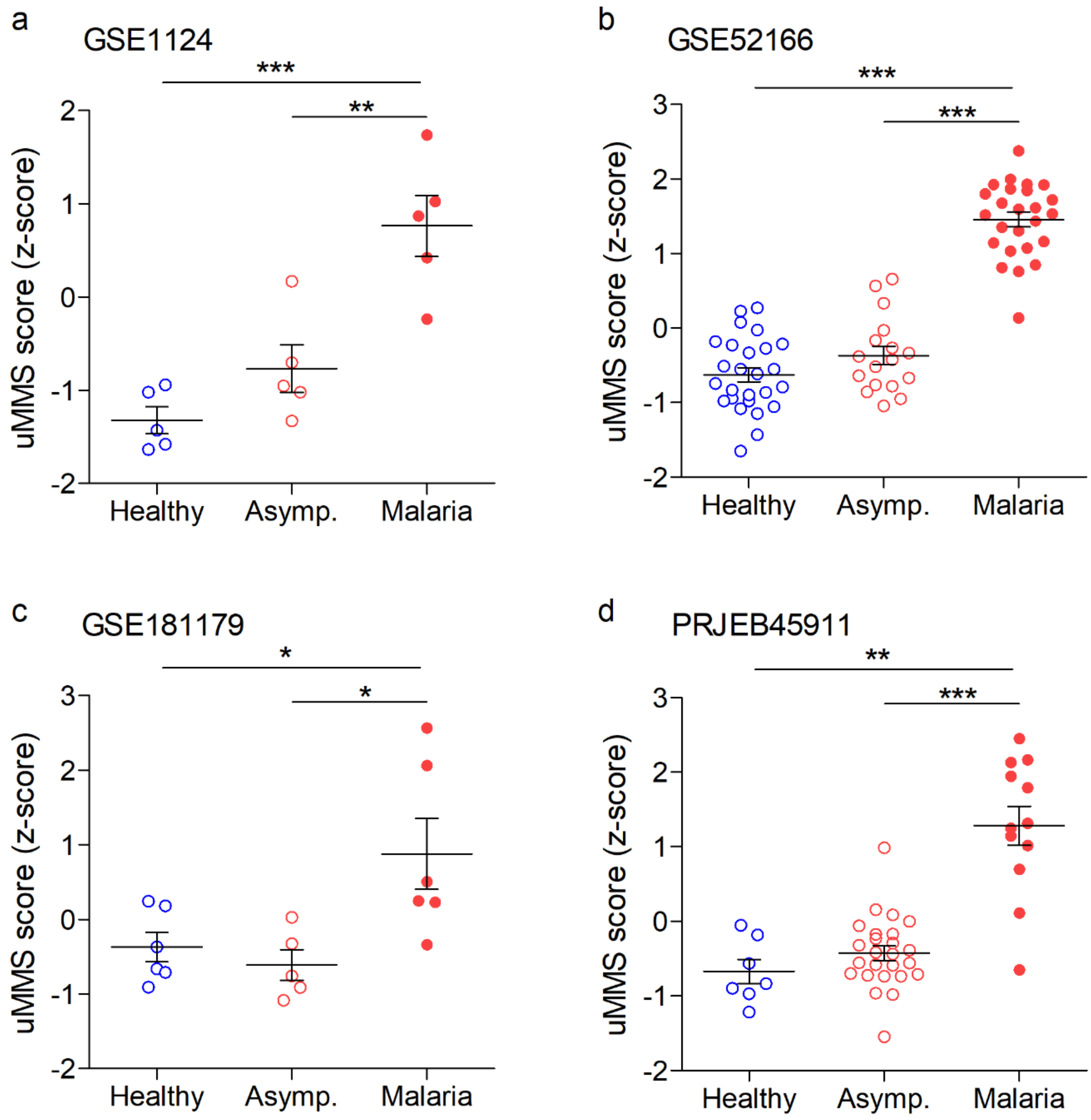


**Supplementary Figure 2 –The uMMS differs between uncomplicated malaria and asymptomatic infection.** Comparison of uMMS scores between healthy controls, individuals with asymptomatic *P. falciparum* infection (Asymp) and patients with uncomplicated malaria (UM) from the datasets GSE1124 (a), GSE52166 (b), GSE181179 (c), and PRJEB45911 (d). Data were analysed with ANOVA followed by Bonferroni multi-comparison test. Statistical significance is shown as *$p < 0.05$, **$p < 0.01$, and ***$p < 0.001$. Error bars represent mean $\pm$ SEM.

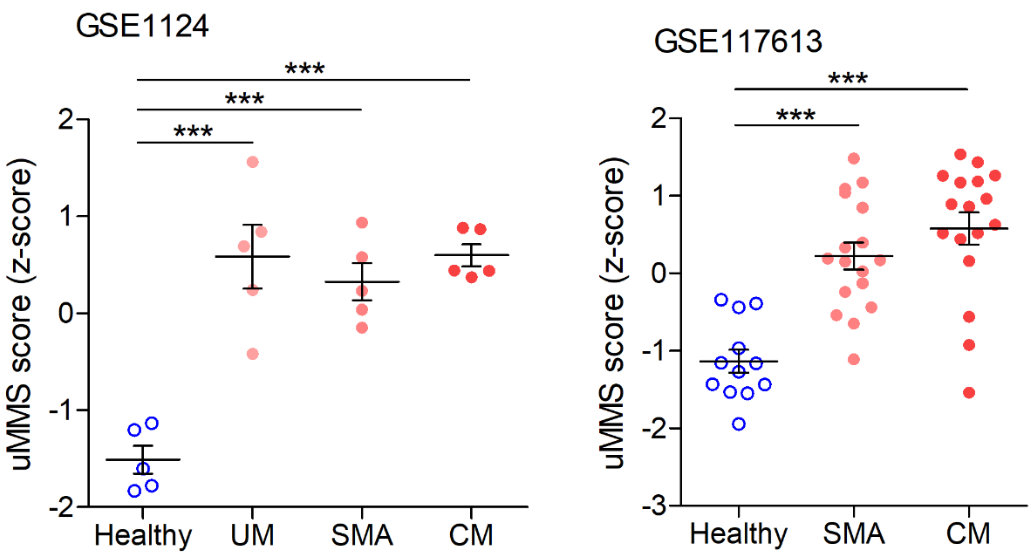


**Supplementary Figure 3 –The uMMS does not differ between uncomplicated, severe malarial anemia and cerebral malaria.** Comparison of uMMS scores between healthy controls, and patients with uncomplicated malaria (UM), severe malarial anemia (SMA), and cerebral malaria (CM) from the datasets GSE1124 and GSE117613. Data were analysed with ANOVA followed by Bonferroni multi-comparison test. Statistical significance is shown as ***p < 0.001. Error bars represent mean ± SEM.

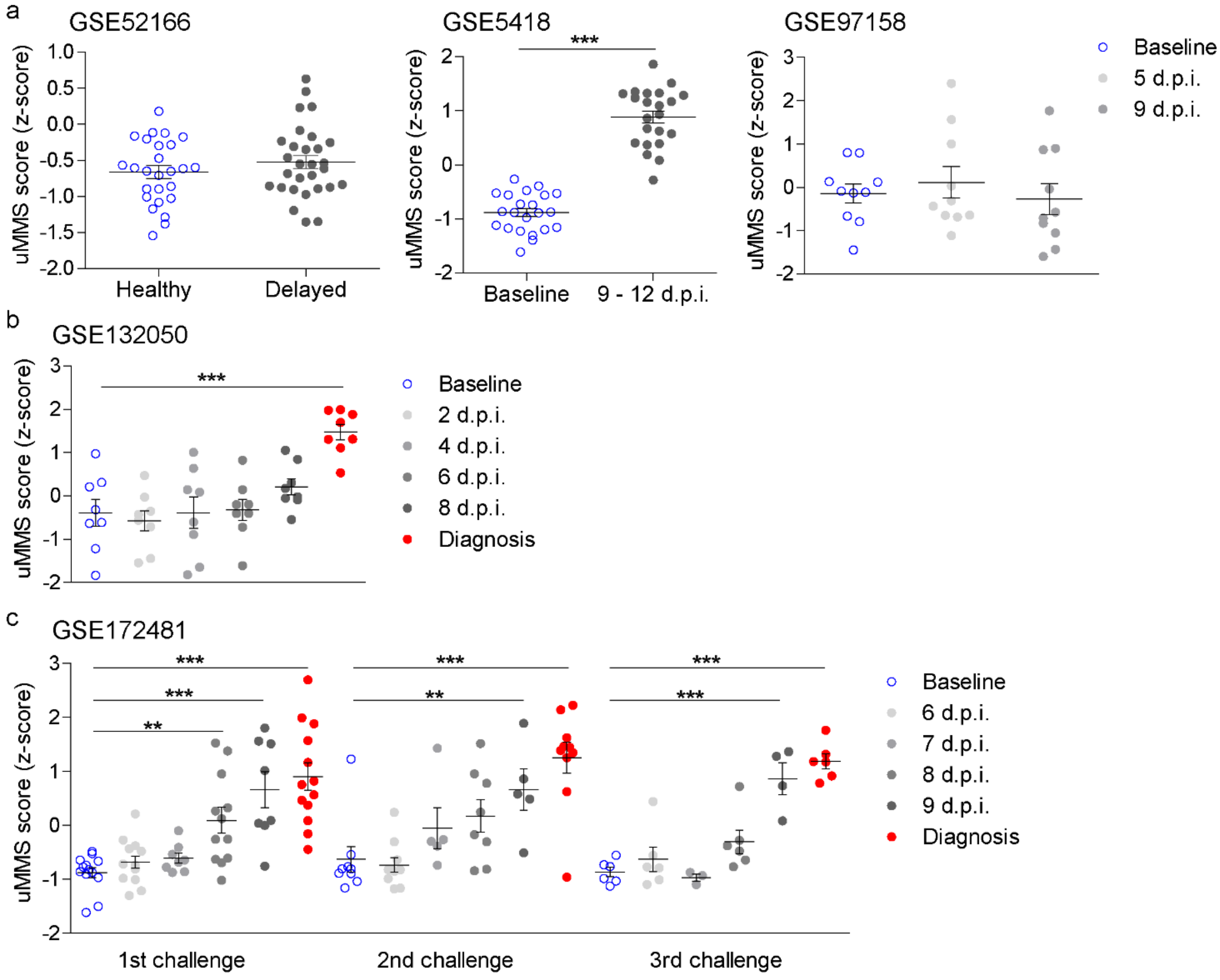


**Supplementary Figure 4 –The uMMS discriminates mixed asymptomatic and symptomatic uncomplicated malaria.** Comparison of uMMS scores between healthy controls and individuals with pre-symptomatic (a - c) and mixed asymptomatic / symptomatic *P. falciparum* infection (b – c). The datasets GSE5418, GSE97158, GSE132050 and GSE172481 are from controlled human malaria infection (CHMI). Diagnosis for datasets GSE132050 and GSE172481 included both asymptomatic individuals with parasitemia above a threshold and/or symptomatic individuals. Data were analysed with ANOVA followed by Bonferroni multi-comparison test. Statistical significance is shown as **p < 0.01, ***p < 0.001. Error bars represent mean ± SEM. d.p.i. = days post-infection.

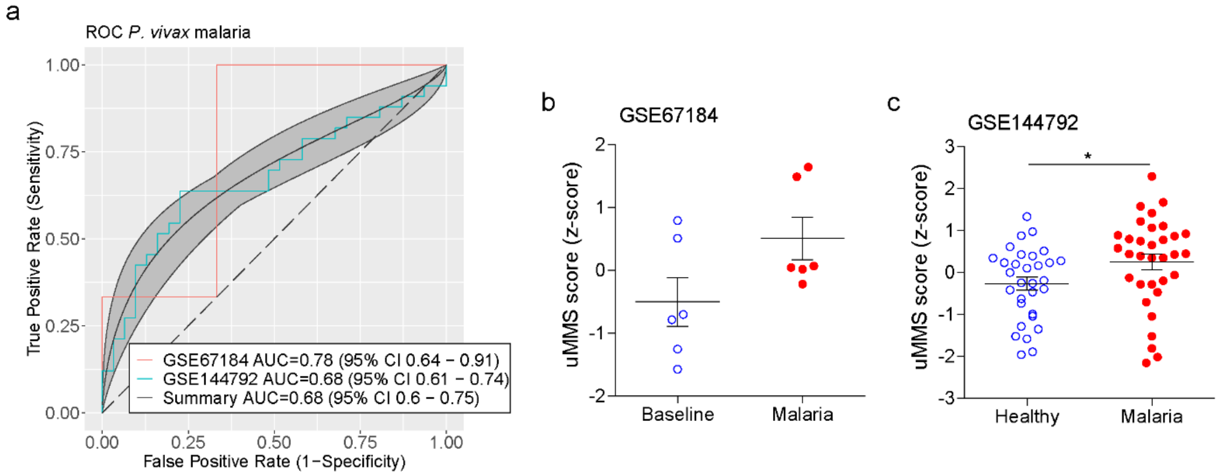


**Supplementary Figure 5 –The uMMS displays lower performance to discriminate *P. vivax* malaria.** (a) ROC curves comparing individuals with *P. vivax* malaria and controls. (b - c) Comparison of uMMS scores between controls and individuals with *P.* vivax malaria in the datasets GSE67184 (b) and GSE144792 (c). Data were analysed with t test. Statistical significance is shown as *p < 0.05. Error bars represent mean ± SEM.

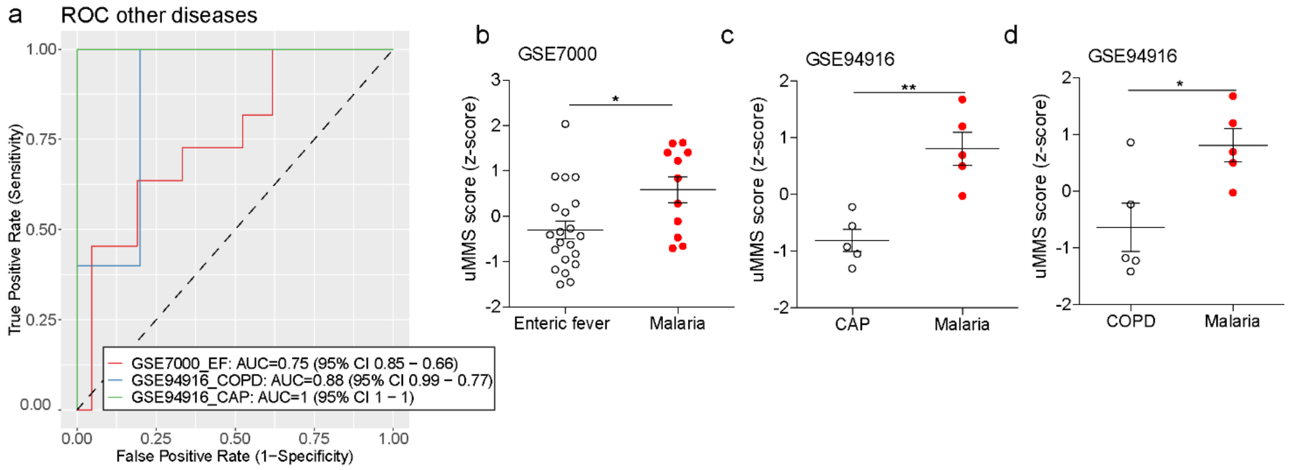


**Supplementary Figure 6 –The uMMS displays moderate performance to discriminate uncomplicated malaria from other diseases.** (a) ROC curves comparing individuals with uncomplicated malaria (UM) and enteric fever (EF), community acquired pneumonia (CAP) and chronic obstructive pulmonary disease (COPD). (b - d) Comparison of uMMS scores between UM and EF (b), CAP (c), and COPD (d). Data were analysed with t test. Statistical significance is shown as *p < 0.05. Error bars represent mean ± SEM.

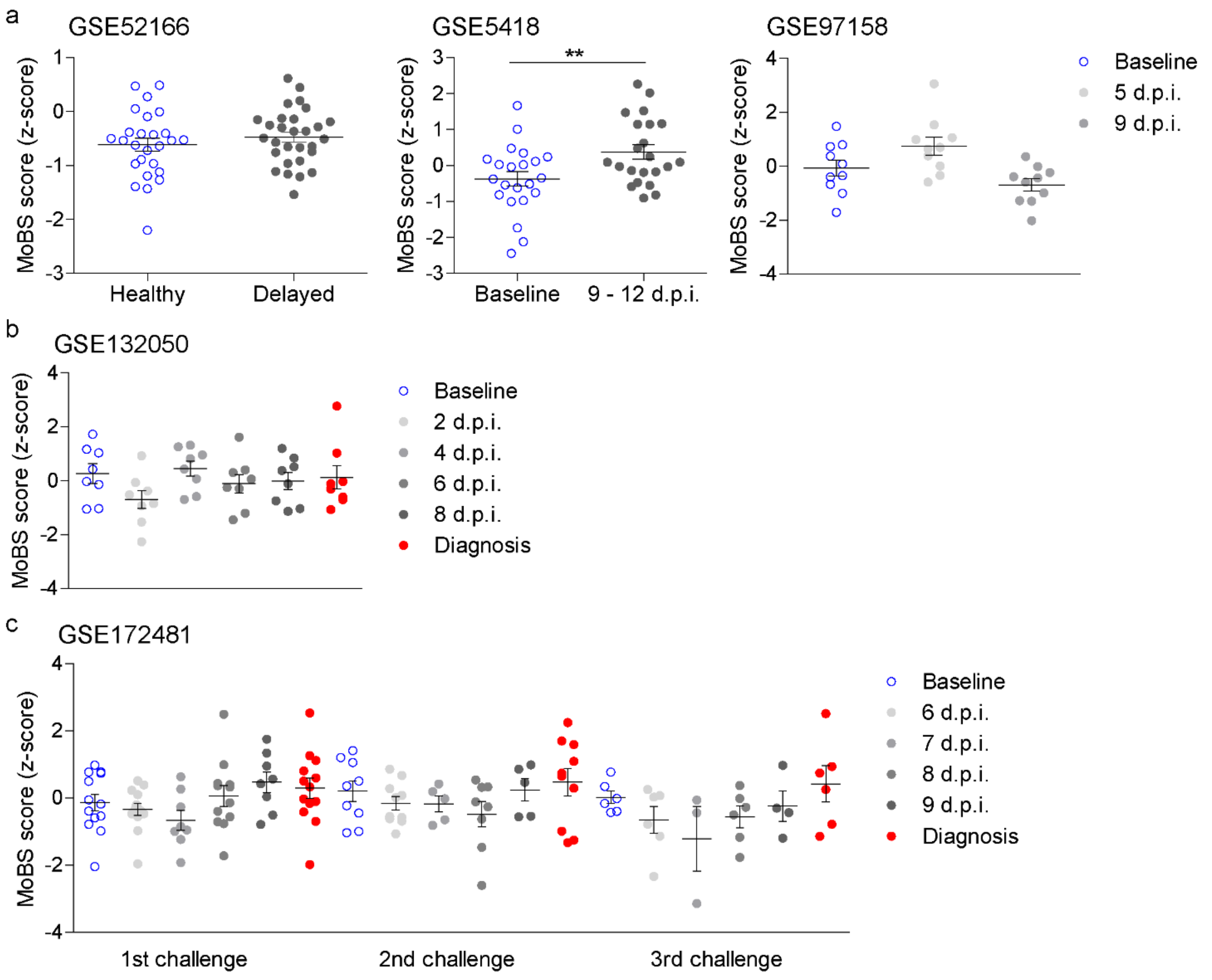


**Supplementary Figure 7 –The Malaria or Bacteria Signature (MoBS) does not discriminate pre-symptomatic or mixed asymptomatic and symptomatic cases.** Comparison of uMMS scores between healthy controls and individuals with pre-symptomatic (a - c) and mixed asymptomatic / symptomatic *P. falciparum* infection (b – c). The datasets GSE5418, GSE97158, GSE132050 and GSE172481 are from controlled human malaria infection (CHMI). Diagnosis for datasets GSE132050 and GSE172481 included both asymptomatic individuals with parasitemia above a threshold and/or symptomatic individuals. Data were analysed with ANOVA followed by Bonferroni multi-comparison test. Statistical significance is shown as **p < 0.01. Error bars represent mean ± SEM. d.p.i. = days post-infection.

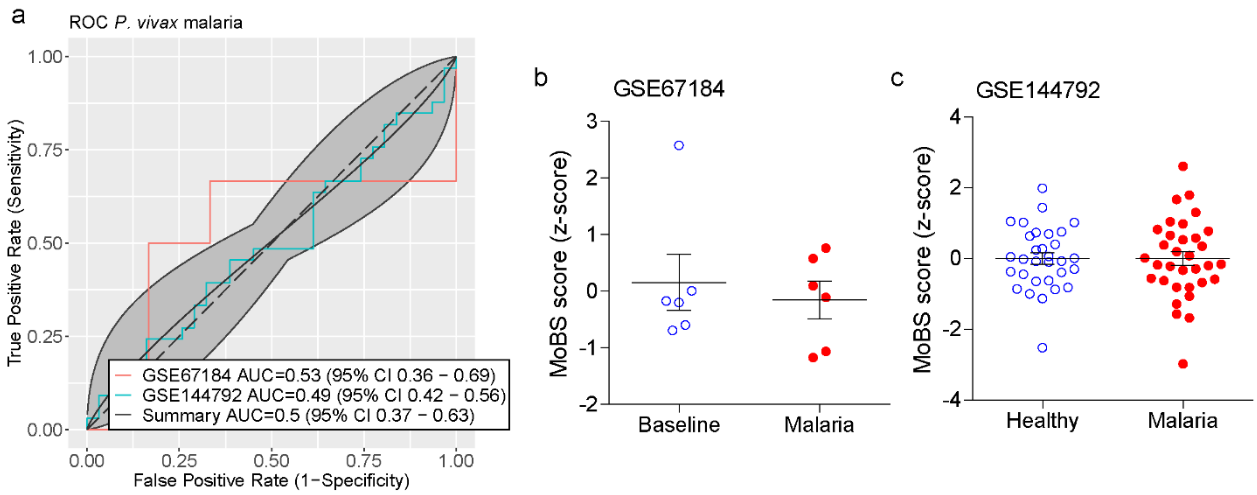


**Supplementary Figure 8 –The MoBS displays poor performance to discriminate *P. vivax* malaria.** (a) ROC curves comparing individuals with *P. vivax* malaria and controls. (b - c) Comparison of MoBS scores between controls and individuals with *P.* vivax malaria in the datasets GSE67184 (b) and GSE144792 (c). Data were analysed with t test. Statistical significance is shown as *p < 0.05. Error bars represent mean ± SEM.

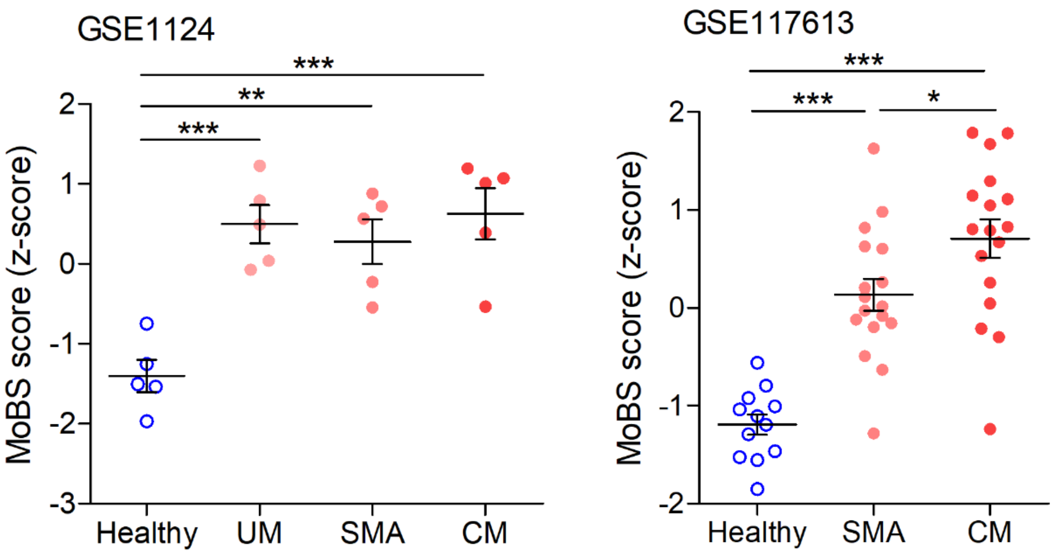


**Supplementary Figure 9 –The MoBS displays poor performance to discriminate uncomplicated malaria, severe malarial anemia and cerebral malaria.** Comparison of MoBS scores between healthy controls, and patients with uncomplicated malaria (UM), severe malarial anemia (SMA), and cerebral malaria (CM) from the datasets GSE1124 and GSE117613. Data were analysed with ANOVA followed by Bonferroni multi-comparison test. Statistical significance is shown as *p < 0.05, **p < 0.01, ***p < 0.001. Error bars represent mean ± SEM.

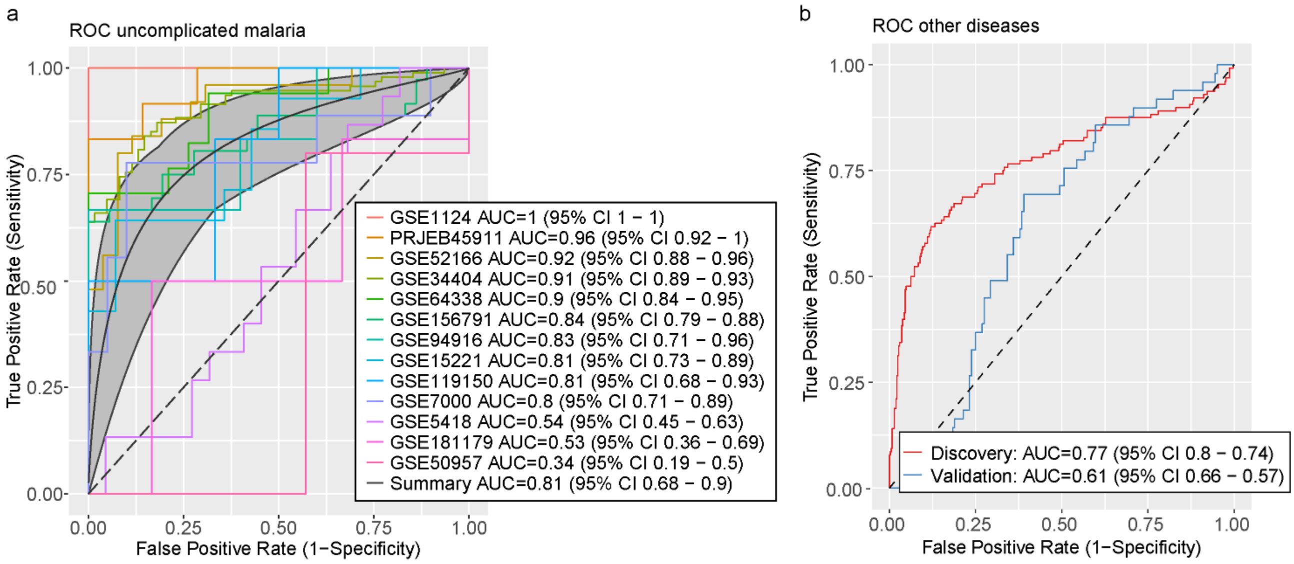


**Supplementary Figure 10 – The cerebral Malaria Meta-Signature (cMMS) has lower generalizability.** (a) ROC curves comparing individuals with uncomplicated malaria (UM) and controls. (b) ROC curves comparing individuals with malaria and enteric fever (EF), sepsis and controls.

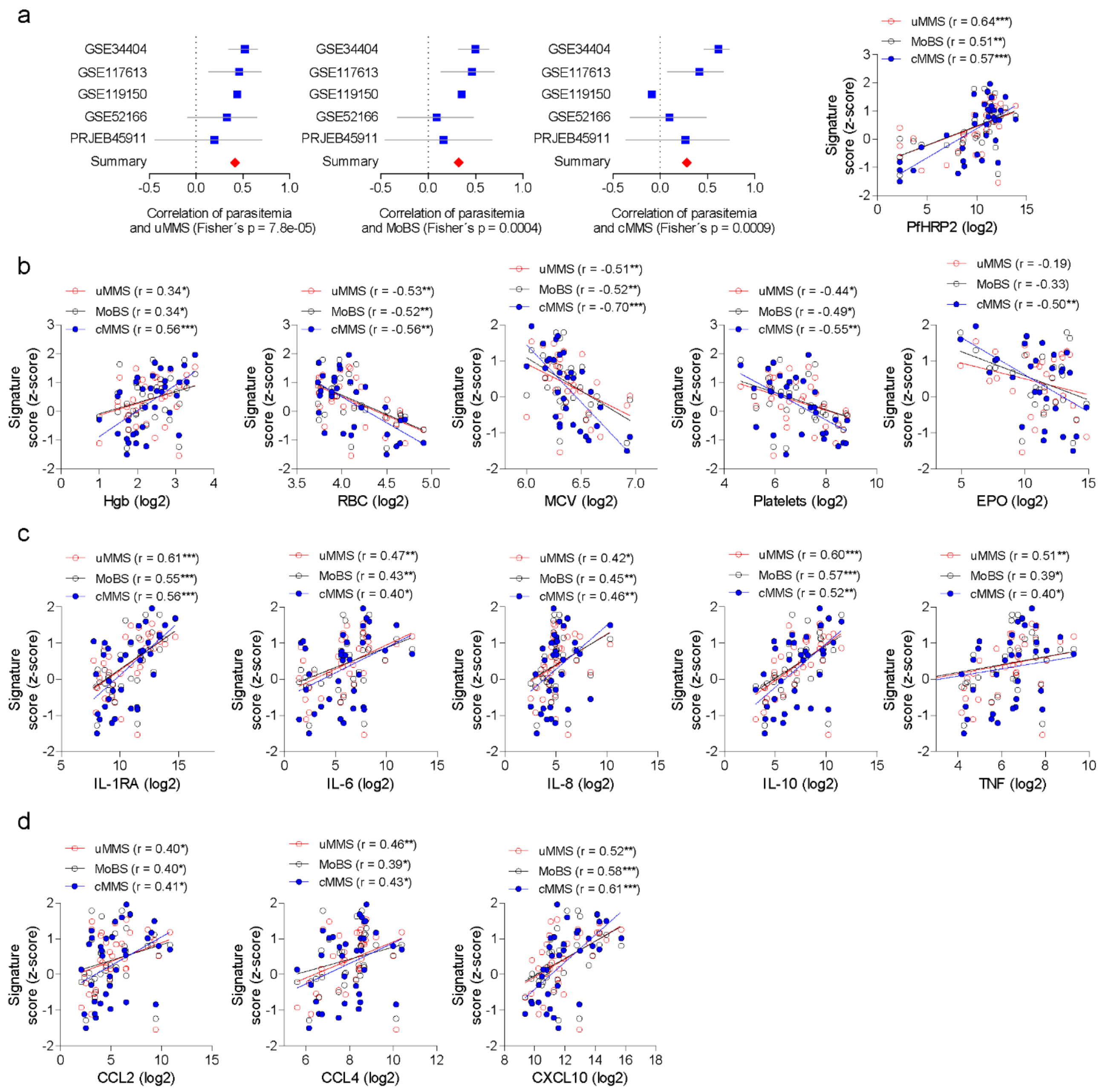


**Supplementary Figure 11 – Gene signature scores correlates with hallmark features of clinical malaria.** (a) Meta-analyses of correlations between parasitemia and uMMS, MoBS, and cMMS scores from datasets GSE117613, GSE34404, GSE119150, GSE52166, and PRJEB45911. Meta-analyses were performed with Fisher´s method. Whiskers represent 95% confidence intervals are represented and the red diamonds reflect the combined Spearman´s correlation coefficient. For GSE117613 we also performed correlations between gene signatures and (a) PfHRP2; (b) hemoglobin (Hgb) leves, platelets and red blood cell (RBC) counts, levels of mean corpuscular volume (MCV) and erythropoietin (EPO); (c) abundance of IL-1RA, IL-6, IL-8, IL-10, TNF, CCL2, CCL4 and CXCL10

in the plasma. Associations were analyzed with Spearman´s rank correlation and the significance are shown as *p < 0.05, **p < 0.01, ***p < 0.001.

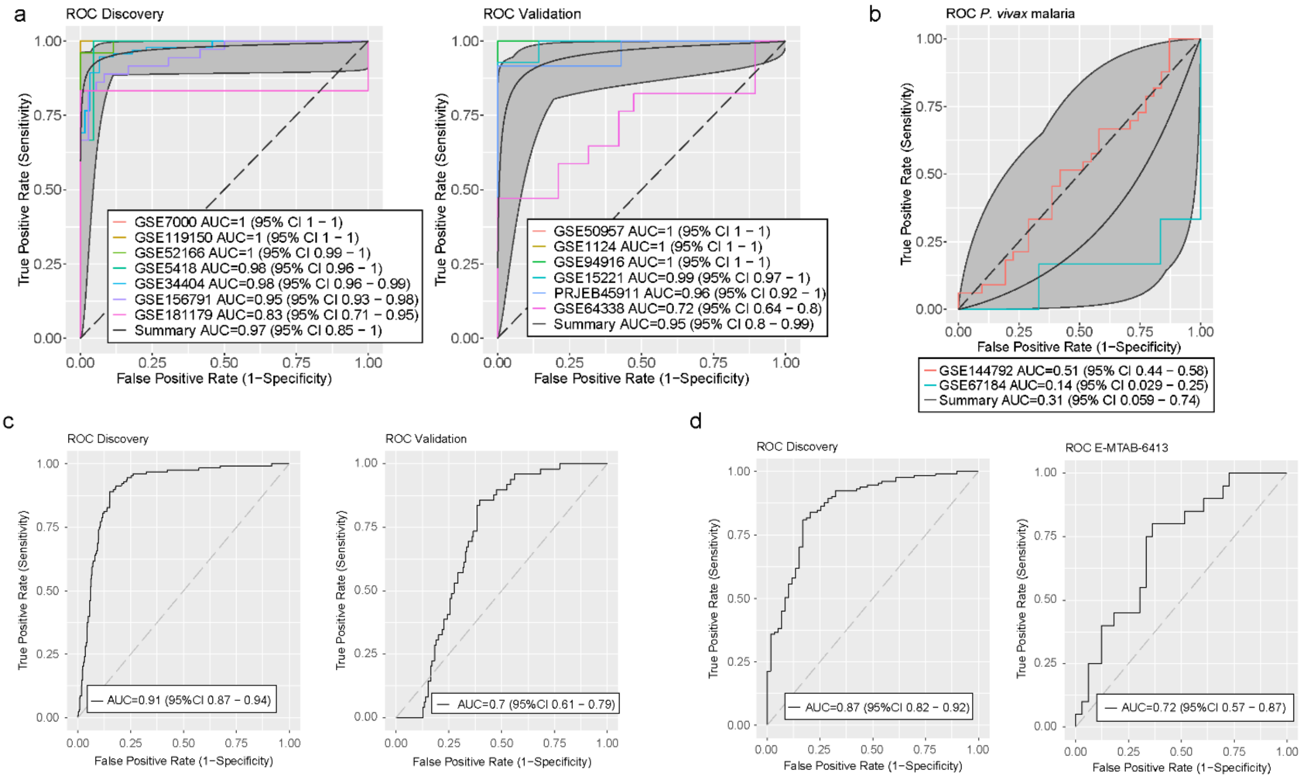


**Supplementary Figure 12 – Discriminatory BTM signatures.** (a) ROC curves comparing individuals with uncomplicated *P. falciparum* malaria (UM) and controls in discovery and validation cohorts using BTM signature identified through meta-analyses. (b) ROC curves comparing individuals with *P. vivax* malaria and controls in two cohorts using BTM signature identified through meta-analyses. (c) ROC curves comparing individuals with malaria and enteric fever (EF), sepsis and controls in discovery and validation cohorts using BTM signature identified through random forest analysis. (d) ROC curves comparing individuals with cerebral malaria (CM) and uncomplicated malaria (UM), severe malarial anemia (SMA), and controls in discovery and validation cohorts using BTM signature identified through random forest analysis.

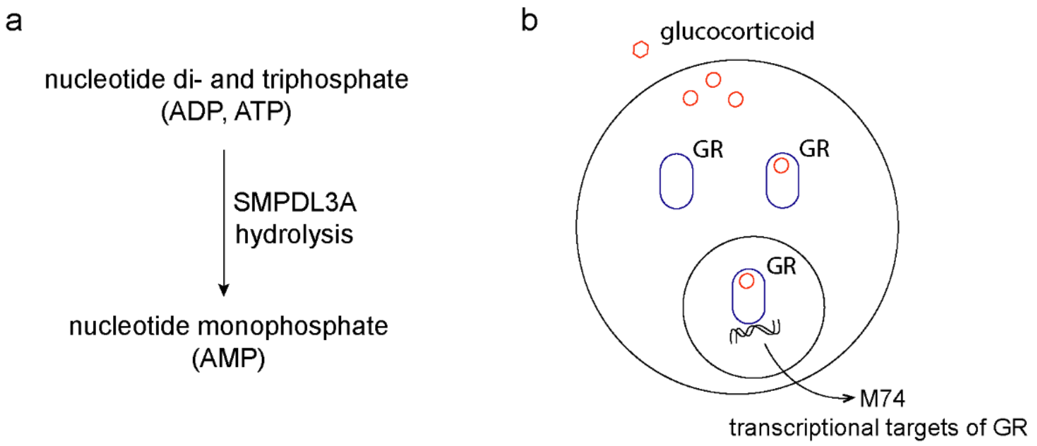


**Supplementary Figure 13 – Functional schematics of SMPDL3A and glucocorticoid receptor.** (a) Schematics of the enzymatic activity of sphingomyelin phosphodiesterase-like 3A (SMPDL3A). [27] (b) Schematics of glucocorticoid receptor (GR) activation and function [28].

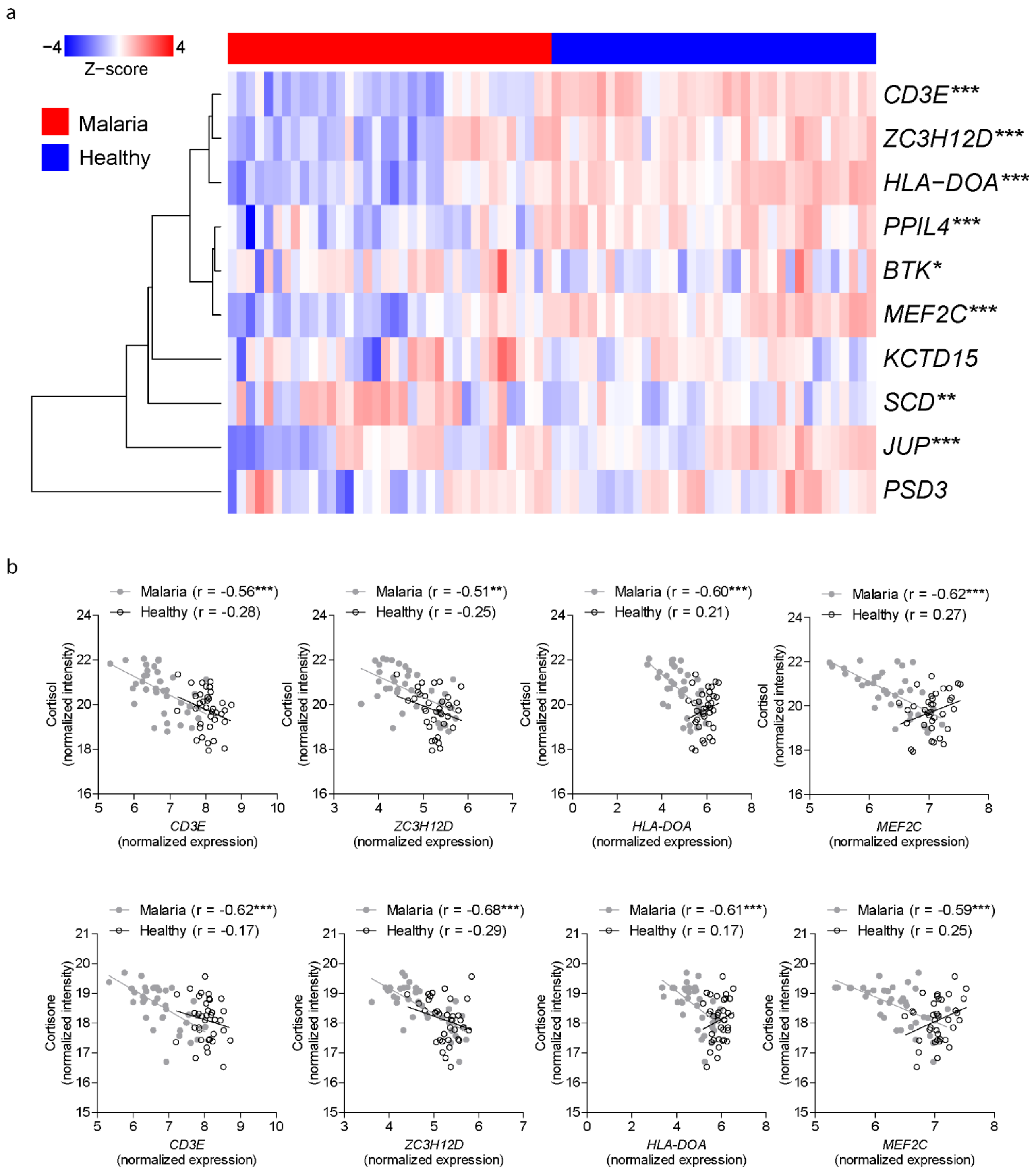


**Supplementary Figure 14 – Glucocorticoid associations with transcriptional targets of glucocorticoid receptor.** (a) Heatmap of member genes from Transcriptional targets of glucocorticoid receptor (M74) before (healthy) and during *P. falciparum* malaria. (b) Correlation between member M74 genes´ expression in blood and abundance of cortisol and cortisone in plasma before (healthy) and during *P. falciparum* malaria. Data were derived from dataset GSE156791 and analyzed with paired t test and Spearman´s rank correlation. Significance is shown as * $p < 0.05$, ** $p < 0.01$, and *** $p < 0.001$.

References


1. Idaghdour Y, Quinlan J, Goulet J-P, et al. Evidence for additive and interaction effects of host genotype and infection in malaria. Proc Natl Acad Sci U S A. **2012**; 109(42):16786–16793.

2. Ockenhouse CF, Hu W, Kester KE, et al. Common and divergent immune response signaling pathways discovered in peripheral blood mononuclear cell gene expression patterns in presymptomatic and clinically apparent malaria. Infect Immun. **2006**; 74(10):5561–5573.

3. Thompson LJ, Dunstan SJ, Dolecek C, et al. Transcriptional response in the peripheral blood of patients infected with Salmonella enterica serovar Typhi. Proceedings of the National Academy of Sciences. Proc Natl Acad Sci U S A. **2009**; 106(52):22433–22438.

4. Li J-J, Huang M-J, Li Z, et al. Identification of potential whole blood MicroRNA biomarkers for the blood stage of adult imported falciparum malaria through integrated mRNA and miRNA expression profiling. Biochem Biophys Res Commun. **2018**; 506(3):471–477.

5. Tran TM, Guha R, Portugal S, et al. A Molecular Signature in Blood Reveals a Role for p53 in Regulating Malaria-Induced Inflammation. Immunity. **2019**; 51(4):750-765.e10.

6. Abdrabou W, Dieng MM, Diawara A, et al. Metabolome modulation of the host adaptive immunity in human malaria. Nat Metab. **2021**; 3(7):1001–1016.

7. Studniberg SI, Ioannidis LJ, Utami RAS, et al. Molecular profiling reveals features of clinical immunity and immunosuppression in asymptomatic P. falciparum malaria. Mol Syst Biol. **2022**; 18(4):e10824.

8. Boldt ABW, Tong H van, Grobusch MP, et al. The blood transcriptome of childhood malaria. EBioMedicine. **2019**; 40:614–625.

9. Subramaniam KS, Spaulding E, Ivan E, et al. The T-Cell Inhibitory Molecule Butyrophilin-Like 2 Is Up-regulated in Mild Plasmodium falciparum Infection and Is Protective During Experimental Cerebral Malaria. J Infect Dis. **2015**; 212(8):1322–1331.

10. Franklin BS, Parroche P, Ataíde MA, et al. Malaria primes the innate immune response due to interferon-gamma induced enhancement of toll-like receptor expression and function. Proc Natl Acad Sci U S A. **2009**; 106(14):5789–5794.

11. Bertrams W, Griss K, Han M, et al. Transcriptional analysis identifies potential biomarkers and molecular regulators in acute malaria infection. Life Sci. **2021**; 270:119158.

12. Tran TM, Jones MB, Ongoiba A, et al. Transcriptomic evidence for modulation of host inflammatory responses during febrile Plasmodium falciparum malaria. Sci Rep. **2016**; 6(1):31291.

13. Prah DA, Dunican C, Amoah LE, et al. Asymptomatic Plasmodium falciparum infection evades triggering a host transcriptomic response. J Infect. **2023**; 87(3):259–262.

14. Milne K, Ivens A, Reid AJ, et al. Mapping immune variation and var gene switching in naive hosts infected with Plasmodium falciparum. Elife. **2021**; 10:e62800.

15. Rothen J, Murie C, Carnes J, et al. Whole blood transcriptome changes following controlled human malaria infection in malaria pre-exposed volunteers correlate with parasite prepatent period. PLoS One. **2018**; 13(6):e0199392.

16. Sandoval DM, Bach F, Nahrendorf W, et al. Adaptive T cells regulate disease tolerance in human malaria [Internet]. medRxiv; 2021 [cited 2023 Apr 13]. p. 2021.08.19.21262298. Available from: https://www.medrxiv.org/content/10.1101/2021.08.19.21262298v1

17. Rojas-Peña ML, Vallejo A, Herrera S, Gibson G, Arévalo-Herrera M. Transcription Profiling of Malaria-Naïve and Semi-immune Colombian Volunteers in a Plasmodium vivax Sporozoite Challenge. PLoS Negl Trop Dis. **2015**; 9(8):e0003978.

18. Easton AV, Raciny-Aleman M, Liu V, et al. Immune Response and Microbiota Profiles during Coinfection with Plasmodium vivax and Soil-Transmitted Helminths. mBio **2020** 11(5):e01705-20.

19. Nallandhighal S, Park GS, Ho Y-Y, Opoka RO, John CC, Tran TM. Whole-Blood Transcriptional Signatures Composed of Erythropoietic and NRF2-Regulated Genes Differ Between Cerebral Malaria and Severe Malarial Anemia. J Infect Dis. **2019**; 219(1):154–164.

20. Dickinson P, Smith CL, Forster T, et al. Whole blood gene expression profiling of neonates with confirmed bacterial sepsis. Genom Data. **2015**; 3:41–48.

21. Blohmke CJ, Muller J, Gibani MM, et al. Diagnostic host gene signature for distinguishing enteric fever from other febrile diseases. EMBO Mol Med. **2019**; 11(10):e10431.

22. Ahn SH, Tsalik EL, Cyr DD, et al. Gene expression-based classifiers identify Staphylococcus aureus infection in mice and humans. PLoS One. **2013**; 8(1):e48979.

23. Thiam A, Sanka M, Ndiaye Diallo R, et al. Gene expression profiling in blood from cerebral malaria patients and mild malaria patients living in Senegal. BMC Med Genomics. **2019**; 12(1):148.

24. Feintuch CM, Saidi A, Seydel K, et al. Activated Neutrophils Are Associated with Pediatric Cerebral Malaria Vasculopathy in Malawian Children. mBio. **2016**; 7(1):e01300-01315.

25. Krupka M, Seydel K, Feintuch CM, et al. Mild Plasmodium falciparum malaria following an episode of severe malaria is associated with induction of the interferon pathway in Malawian children. Infect Immun. **2012**; 80(3):1150–1155.

26. Lee HJ, Georgiadou A, Walther M, et al. Integrated pathogen load and dual transcriptome analysis of systemic host-pathogen interactions in severe malaria. Sci Transl Med. **2018**; 10(447).

27. Traini M, Quinn CM, Sandoval C, et al. Sphingomyelin Phosphodiesterase Acid-like 3A (SMPDL3A) Is a Novel Nucleotide Phosphodiesterase Regulated by Cholesterol in Human Macrophages. J Biol Chem. **2014**; 289(47):32895–32913.

28. Oakley RH, Cidlowski JA. The Biology of the Glucocorticoid Receptor: New Signaling Mechanisms in Health and Disease. J Allergy Clin Immunol. **2013**; 132(5):1033–1044.
